# Supplementary material for: Social prescribing: less rhetoric and more reality. A systematic review of the evidence
Source: BMJ Open. 2017 Apr 6;7(4):e013384. doi: 10.1136/bmjopen-2016-013384 (PMC5558801; doi:10.1136/bmjopen-2016-013384)
Supplement: supplementary appendix [file bmjopen-2016-013384supp001.pdf]

Appendix 1: Search strategies

ASSIA via Proquest Search date 26th June 2015 and 5th February 2016

"social prescrib*" OR "social prescrip*" OR "community referral*"

CINAHL via EBSCO search date 26th June 2015 and 5th February 2016

social prescribing OR "social prescrip*" OR "community referral*"

Ovid MEDLINE(R) In-Process & Other Non-Indexed Citations and Ovid MEDLINE(R) <1946 to Present> searched 26th June 2015 and 5th February 2016

1 social prescrib$.ti,ab.
2 social prescrip$.ti,ab.
3 community referral$.ti,ab.
4 non-medical referral$.ti,ab.
5 well being program$.ti,ab.
6 well-being program$.ti,ab.
7 wellbeing program$.ti,ab.
8 1 or 2 or 3 or 4 or 5 or 6 or 7

Social Care Online via http://www.scie-socialcareonline.org.uk/ searched 26th June 2015 and 5th February 2016

"Social prescribing" OR "social prescription*" or "community referral*"

Social Policy & Practice via OVID search date 26th June 2015 and 5th February 2016

1 social prescrib$.ti,ab.
2 social prescrip$.ti,ab.
3 community referral$.ti,ab.
4 non-medical referral$.ti,ab.
5 well being program$.ti,ab.
6 well-being program$.ti,ab.
7 wellbeing program$.ti,ab.
8 1 or 2 or 3 or 4 or 5 or 6 or 7

Google search last performed 5th January 2016

Two reviewers independently searched google.co.uk using the search terms "social prescribing" and "community referral" and reviewed the search results from the first 10 pages
