# Supplementary material for: Social prescribing: less rhetoric and more reality. A systematic review of the evidence
Source: BMJ Open. 2017 Apr 6;7(4):e013384. doi: 10.1136/bmjopen-2016-013384 (PMC5558801; doi:10.1136/bmjopen-2016-013384)
Supplement: Author's manuscript [file bmjopen-2016-013384.draft_revisions.pdf]

BMJ Open

Social prescribing: less rhetoric and more reality. A systematic review of the evidence

Journal:	*BMJ Open*
Manuscript ID	bmjopen-2016-013384
Article Type:	Research
Date Submitted by the Author:	08-Jul-2016

Complete List of Authors:	Bickerdike, Liz; University of York, Centre for Reviews and Dissemination<br>Booth, Alison; University of York, Dept of Health Sciences<br>Wilson, Paul; University of Manchester, Alliance Manchester Business School<br>Farley, Kate; University of Leeds, School of Healthcare<br>Wright, Kath; University of York, Centre for Reviews & Dissemination

**Primary Subject Heading**:	Health services research
Secondary Subject Heading:	General practice / Family practice
Keywords:	Health policy < HEALTH SERVICES ADMINISTRATION & MANAGEMENT, Organisation of health services < HEALTH SERVICES ADMINISTRATION & MANAGEMENT, PRIMARY CARE

SCHOLARONE™
Manuscripts

For peer review only

Social prescribing: less rhetoric and more reality. A systematic review of the evidence

Liz Bickerdike[1], Alison Booth[2], Paul M Wilson[3], Kate Farley[4], Kath Wright[1]

1. Centre for Reviews and Dissemination, University of York
2. York Trials Unit, University of York
3. Alliance Manchester Business School, University of Manchester
4. School of Healthcare, University of Leeds

Address for correspondence

Paul Wilson
Alliance Manchester Business School
University of Manchester
Booth Street East
Manchester
M15 6PB

Email: paul.wilson@manchester.ac.uk

For peer review only

For peer review only - http://bmjopen.bmj.com/site/about/guidelines.xhtml

Objectives: Social prescribing is being widely promoted and adopted as means of alleviating some of the pressures on general practice by supporting people access to services that can help improve their health and well-being. We conducted a systematic review to assess the evidence for the effectiveness of social prescribing programmes relevant to the UK NHS setting.

Setting/data sources: Nine databases were searched from 2000 to January 2016 for studies conducted in the UK. Relevant reports and guidelines, websites and reference lists of retrieved articles were scanned to identify additional studies. All the searches were restricted to English language only.

Participants: Systematic reviews and any formal evaluation of programmes where referral was made from a primary care setting to a link-worker or facilitator of social prescribing were eligible for inclusion. Risk of bias for included studies was undertaken independently by two reviewers and a narrative synthesis was performed.

Primary and secondary outcome measures: Primary outcomes of interest were any measures of health and wellbeing and or utilisation of health services.

Results: We included a total of 15 evaluations of social prescribing programmes. Most were small scale and limited by poor design and reporting. All were rated as a having a high risk of bias. Common design issues included a lack of comparative controls, short follow up durations, a lack of standardised and validated measuring tools, missing data and a failure to consider potential confounding factors. Despite clear methodological shortcomings, most evaluations presented positive conclusions.

Conclusions: Social prescribing is being widely advocated and implemented but current evidence fails to provide sufficient detail to judge either success or value for money. If social prescribing is to realise its potential, future evaluations must be comparative by design and consider when, for whom, how well and at what cost.

Trial registration: PROSPERO Registration: CRD42015023501

For peer review only

For peer review only - http://bmjopen.bmj.com/site/about/guidelines.xhtml

Strengths and limitations

Social prescribing is being widely promoted and adopted as means of dealing with some of the pressures on general practice. It provides GPs with a way of helping people access sources of support within the community to help improve their health and well-being.

In our review, we identified 15 evaluations but found little convincing evidence for either effectiveness or value for money; most evaluations were small scale pilot projects limited by poor design and reporting.

Despite these shortcomings, most projects have presented positive conclusions, generating a momentum for social prescribing that does not appear to be supported by the research evidence.

If social prescribing is to realise its potential, future evaluations must be comparative by design and consider when, for whom, how well and at what cost.

For peer review only

For peer review only - http://bmjopen.bmj.com/site/about/guidelines.xhtml

Background

With estimates of a £30 billion funding gap by 2020, a radical rethink of the way health services are currently delivered remains high on the policy agenda. The Five Year Forward View has stressed that developing innovative approaches to delivering health care are integral to the long term future of the National Health Service (NHS).[1]

Social prescribing is one such model and is being widely promoted as a way of making general practice more sustainable. Social prescribing is a way of linking patients in primary care with sources of support within the community. It provides GPs with a non-medical referral option that can operate alongside existing treatments to improve health and well-being. There is no widely agreed definition of social prescribing but schemes usually involve the referral of patients to a link worker, to co-design a nonclinical social prescription to improve their health and wellbeing, commonly using services provided by the voluntary and community sector.[2] This can include an extensive range of practical information and advice, community activity, physical activities, befriending, and enabling. The types of activities offered as part of a social prescribing service can aim to help address the psychological problems and low levels of wellbeing often manifest in frequent attenders in general practice. By addressing these it is often hoped that there will be a subsequent positive impact on frequency of attendance.[3]

The Department of Health have advocated the introduction of social prescriptions for those with long-term conditions,[4] and NHS England have announced the appointment of a national clinical champion for social prescribing.[5] With the current Secretary of State for Health also promoting access to non-clinical interventions that take a more 'holistic view',[1 6] support for social prescribing is significant at the policy level

Many localities are now offering or considering implementing social prescribing programmes, but is the apparent enthusiasm justified? As part of a study which aimed to help NHS commissioners make better use of research in their decision making,[7] we examined the evidence for social prescribing. This systematic review summarises the evidence for the effectiveness of social prescribing programmes relevant to the UK NHS setting.

For peer review only

Methods

The protocol and amendments were registered in PROSPERO (Registration number: CRD42015023501).

Data sources and searches

DARE, Cochrane Database of Systematic Reviews and NHS EED were searched for relevant systematic reviews and economic evaluations (24th June 2015; no new records added to DARE and NHS EED databases from January 2015 so we did not run updated searches).

We searched the following databases (initial search 26th June 2015; updated search 5th February 2016): ASSIA, CINAHL, MEDLINE, Social Care Online and Social Policy & Practice.

NICE, SCIE and NHS Evidence were searched for reviews, guidance, evidence briefings or any other papers describing or evaluating social prescribing programmes. We searched Google to identify grey literature reports of relevant evaluations in UK settings (5th January 2016). Additional searches of specific organizational websites such as the Kings Fund, Health Foundation, Nuffield Trust and NESTA were also undertaken. Reference lists of retrieved articles were scanned to identify additional studies.

All the searches were restricted to English language only and published between 2000 to January 2016. The search strategies are available in Appendix 1.

Study selection

Systematic reviews and any formal evaluations of social prescribing programmes being delivered within a primary care setting were eligible for inclusion. Studies were eligible regardless of whether a comparison group was included. Primary outcomes of interest were any measures of health and wellbeing, including self-reported measures (for example levels of physical activity or depression scores). We also considered any other outcomes used in the included evaluations.

We included only studies where referral was made from a primary care setting to a co-ordinator, link-worker or facilitator of social prescribing (this type of role will be referred to as "link-worker" throughout this review). Any interventions being specifically delivered as part of a social prescribing programme were included in the review.

We excluded studies where referral was made outside of a primary care setting [8] and any social prescribing interventions delivered as part of mental health or counselling services such

For peer review only - http://bmjopen.bmj.com/site/about/guidelines.xhtml

as an Improving Access to Psychological Therapies (IAPT) programme. We also excluded evaluations of activities that could be socially prescribed (for example physical activity programmes or community arts projects) but did not involve referral to a link-worker in the first instance. [9-12]

Study selection was performed by one researcher and checked by a second, with any discrepancies resolved by discussion or a third reviewer.

Data extraction and quality assessment

Details of the setting, participants, the intervention (type, delivery mode and length of time), type of evaluation and outcomes of evaluation were extracted and quality assessed by one researcher and checked by a second. Discrepancies were resolved by discussion or by recourse to a third researcher.

We used the Cochrane risk of bias tool to assess the quality of the randomised controlled trial.[13] To assess the quality of the before and after evaluations we applied the quality assessment tool developed by the US National Heart, Lung and Blood Institute for before-after (pre-post) studies with no control group.[14]

Data synthesis and analysis

We performed a narrative synthesis of the evidence. There was insufficient data to perform meta-analysis for any of the outcomes of interest. No subgroup analyses were planned.

For peer review only - http://bmjopen.bmj.com/site/about/guidelines.xhtml

Results

We identified a total of 431 records through database searching and a further 14 records through other sources. After deduplication 341 titles and abstracts were screened and 70 full text papers were assessed for inclusion (see Figure 1: PRISMA flow diagram).

Excluded studies

We excluded 45 studies on eligibility grounds and were unable to access the full text for seven identified records. We also identified two non-systematic reviews of social prescribing schemes.[15 16] These were excluded as they did not critically appraise included studies and were limited in their synthesis of findings; one review included a number of evaluations that did not meet our inclusion criteria.[16] We checked the reference lists of both reviews to ensure we had identified and included all relevant evaluations.

Included studies

We included a total of 15 evaluations (reported in 16 papers) of social prescribing programmes where some form of link-worker role was utilised.[3 17-31] The designs included one RCT,[17] one non-RCT,[18] two qualitative studies,[22 27] four uncontrolled before and after studies,[3 19-21] and eight descriptive reports of six evaluations, of which five included some analysis of qualitative data.[23-26 28-31] Details of the included evaluations are presented in Table 1.

In each of the included studies, the link-worker (job title variously named) met with the patient to discuss their needs and directed them to appropriate community/voluntary sector sources of support in their locality. The training and knowledge of people fulfilling these types of link-worker role varied between projects. In some services this was paid role, in others these roles were fulfilled by volunteers. Some link-worker had good knowledge and existing networks with local services in place [27-29] and in others they received some basic training and made use of a directory of resources.[21]

Patients were referred to a range of activities provided by local or national voluntary and community sector organisations. Interventions received included exercise and other physical activities, signposting to housing, welfare and debt advice, adult education and literacy, befriending, counselling, self-help support groups, luncheon clubs and art activities.

The number of referrals made to social prescribing programmes ranged from 30 to 1607. Referrals were made by a range of health professionals but primarily GPs. Three of the studies reported that feedback was given to the referrer about the actions taken and the

For peer review only - http://bmjopen.bmj.com/site/about/guidelines.xhtml

participants' progress in the social prescribing programme.[21 27 29]

Quality of the evidence

In the randomised controlled trial only sequence generation was adjudged to be of low risk of bias; all other criteria were rated as unclear or high risk. [17] The authors reported that the randomisation process was misunderstood in two of the participating practices but random allocation appeared to be maintained. A key inclusion criteria for the Cochrane Effective Practice and Organisation of Care Review Group is that a controlled before and after study must have at least two intervention and two control g to guard against confounding.[32] Here, the controlled before and after study includes one intervention and one control group, drawn from the same general practice. As such, we rated the study as having a high risk of bias and made no further assessment of quality with the Cochrane risk of bias tool. Uncontrolled before-and-after studies are inherently weak evaluative designs and no included study fulfilled all of the specified quality criteria. In general, evaluations had small sample sizes (less than 100 participants), significant loss to follow up (>20%), were lacking in completeness of outcome data and had unclear selection criteria for the study population. Follow-up periods were generally short (immediately post-intervention up to 4 months post-intervention). There is a therefore a high risk of bias.

Uptake and attendance

Seven of the 14 included studies reported the number of people attending an initial appointment with a link-worker. Where, reported attendance at this initial appointment ranged from 50% to 79% of those referred by a primary care professional to a social prescribing programme.[17 20-22 24-26] Participants' attendance at activities to which they were subsequently referred or recommended was reported in only two studies and varied from 58% [21] to 100%.[20]

Health and wellbeing outcomes

The RCT [17], two uncontrolled before-and-after studies [20 21] and three descriptive reports [25 26 31] measured health and wellbeing outcomes at baseline and again at up to 6 months after participation in a social prescribing programme; one study reported outcomes at up 12 months. The measures used were Warwick-Edinburgh Mental Wellbeing Scale (WEMWBS;[20 25 31]), Hospital Anxiety and Depression Scale (HADS;[17]), General Anxiety Disorder-7 (GAD-7;[26]); Patient Health Questionnaire-9 (PHQ-9): [26]); Clinical Outcomes in Routine Evaluation-Outcome Measure (CORE-OM);[21], WSAS ([20 21]), General Health Questionnaire (GHQ-12; [21]) and COOP/WONCA.[17] Table 3 presents findings for studies using validated measures; all report some improvements in health and wellbeing. However it is difficult to

For peer review only - http://bmjopen.bmj.com/site/about/guidelines.xhtml

quantify the size of the observed improvements due to a lack of reported detail, a lack of sufficient control group data, and differences in reporting between studies. It is not possible to determine whether any observed improvements were clinically significant. Studies reported short-term outcomes only; there is no evidence about the effect social prescribing has on health and wellbeing outcomes beyond six months.

One uncontrolled before and after study used a bespoke measure, the Wellspring Wellbeing Questionnaire, comprising PHQ9 and GAD7 tools, and items from ONS's Wellbeing Index/Integrated Household Survey and International Physical Activity Questionnaires.[3] A second also used a bespoke measure which utilised a 5-point scale across eight domains associated with different aspects of self-management such as 'looking after yourself' and 'managing symptoms'.[19] Two further descriptive reports also indicated they used the WEMWBS to measure changes in health and wellbeing but poor reporting and what appears to be very small numbers of responders.[23 24] In the two studies using non-validated measures some positive improvements in outcomes such as depression and anxiety at 3 to 4 months' follow up were reported.[3 19]

Health care utilisation outcomes

Both comparative evaluations[17 18] and three of the uncontrolled before and after studies[3 19 21] reported some measure of health care utilisation; comparing hospital episode statistics (HES) and/or GP record data from 6 to 12 months before intervention with data up to 18 months post intervention. Outcomes included GP consultations, referrals to secondary care, in-patient admissions and A&E attendances. Findings were mixed. The RCT reported that the number of primary care contacts were similar between intervention and control groups; there were fewer referrals to secondary care and more prescription drugs for those in the intervention group compared with the control group.[17] The non-randomised trial reported statistically non-significant reductions in primary care contacts (face-to-face and/or telephone) and referrals to secondary care[18]. The before and after studies reported reductions in secondary care referrals, in-patient admissions and A&E attendances,[19] "significant" reduction in primary care contact,[21] and a decrease in face-to-face GP contact but increase in telephone contact.[3]

Patient experience

Three before and after studies [19-21] and five descriptive reports[22 25 27 29 31] reported patient experience outcomes. Studies used semi-structured interviews or survey questionnaires specifically designed for the project evaluation to assess participant experience.

For peer review only - http://bmjopen.bmj.com/site/about/guidelines.xhtml

In six of the studies participants reported overall satisfaction with social prescribing programmes.[19-21 25 27 29] General improvements in feelings of loneliness and social isolation,[20 29 31] and improved mental and physical health were also observed.[20] Issues that may impact willingness to participate in socially prescribed activities include confidence,[20 29] interest in/appropriateness of activities on offer[20 29] and literacy or travel issues.[29 31] One qualitative study reported that patients had poor knowledge of the service prior to attending their appointment with the link-worker resulting in some participants feeling that the service did not meet their expectations.[22] Another evaluation identified a similar issue regarding a lack of understanding of the service among participants.[31]

Referrer experience and lessons learned

A small number of studies conducted semi-structured interviews with primary care practitioners referring participants to social prescribing programmes and/or link-workers.[20 25 27-31] GPs in general found that being able to make a social prescription was a useful additional tool.[20 27 28 30] Key issues identified for successful implementation of social prescribing programmes were central coordination of referrals,[25] resources and training to support co-ordinators and enabling networking with the voluntary and community sector,[25 28] and good communication between GPs, participants and link-workers: social prescribing is unfamiliar to many GPs and requires good clear explanation to engage participants[20 22 25 31]; delivering feedback on participants' progress encourages GP support for social prescribing.[27 29 30]

Costs

The two comparative evaluations reported costs. One found total mean costs were greater in the intervention group (£153) compared with the control group (£133).[17] The other reported no statistically significant differences between the financial and environmental costs of healthcare use between the intervention and control groups[18].

One before and after study undertook a cost-benefit analysis using estimated input costs and benefits derived from 12 month outcome data obtained for 108 patients referred to social prescribing (42 of whom were referred to funded voluntary and community service providers). A total NHS cost reduction of £552,189 was generated by multiplying the estimated per-patient cost reduction by the total number of referrals (n=1118) to funded voluntary and community service providers of was achieved over the 2 year course of a social prescribing pilot programme. This estimate was compared with total estimated input costs of £1.1 million.[19]

One other report of an evaluation estimated total running costs of £83,144 for the programme for one year.[3].

For peer review only - http://bmjopen.bmj.com/site/about/guidelines.xhtml

Discussion

This systematic review has examined the evidence to inform the commissioning of social prescribing schemes. Overall, we identified 15 evaluations but have found little convincing evidence for either effectiveness or value for money.

Most of the evaluations of social prescribing activity are small scale and limited by poor design and reporting. Missing information has made it difficult to assess who received what, for what duration, with what effect and at what cost. Common design weaknesses include a lack of comparators (increasing the risk of bias), loss to follow up, short follow up durations, a lack of standardised and validated measuring tools and a failure to consider potential confounding factors. This last issue is particularly important as most referred patients appear to have been receiving other interventions and so we have no way of assessing the relative contributions of the interventions to the outcomes reported. Despite these methodological shortcomings most evaluations have presented positive conclusions, generating a momentum for social prescribing that does not appear to be warranted.

Strengths and limitations

Our systematic review appears to be the first to assess the effectiveness of social prescribing programmes relevant to the UK NHS setting. We have searched for full publications and grey literature since 2000 but it is possible that we have not identified some local evaluations. However, we think it unlikely that any unidentified evaluations will be more robust than those included in the review.

Many of the evaluations were written as narrative reports and as such do not adhere to formal reporting standards that would be expected in reports to funding agencies or in academic journal articles. This made extracting relevant data more difficult and it is possible key information may have been missed. Even if this shortcoming of data completeness were to be addressed we believe that it would do little to alter the overall picture of a low quality evidence base at high risk of bias.

Implications

Our systematic review has not established that there is clear evidence that social prescribing does not work. Rather, we are not yet able to reliably judge which if any social prescribing programmes demonstrate a degree of promise and so could be considered further. For those seeking to commission new or extend existing schemes this evidence gap is a hindrance rather than a help, especially so given the widespread support and advocacy for social prescribing at the policy level.

For peer review only - http://bmjopen.bmj.com/site/about/guidelines.xhtml

Whilst the tension between rigour and 'good enough' evidence has long been recognised,[33] even 'good enough' is severely lacking from the social prescribing literature be that in the design or in the conduct of the evaluations themselves. This may in part reflect the way schemes have 'emerged' rather than being systematically planned with evaluation built in from the outset. Nevertheless, if social prescribing is to realise its potential then there is an urgent need to improve the ways by which schemes are evaluated.

Prospective pathways for undertaking rigorous planned experimental evaluation are well defined,[34] but the opportunity, time and resources needed to employ these in a service context can be limited. However, this does not serve as an excuse for inaction and in the current financial climate we should of course only be investing in those services where we can demonstrate real benefit over existing ways of working. What this should mean for future evaluation of social prescribing is that a more coordinated approach to the planning, implementation and evaluation of new and existing schemes is undertaken. This could and should involve the adoption of a common analytical framework which in turn will facilitate standardised metrics, cross-site comparison and shared learning. The IDEAL framework offers one such pathway to navigate the evaluation continuum that would allow for the iterative development and evaluation of whether social prescribing is likely to succeed in a particular setting and allow for adaptation, refinement and system integration without losing sight of the need for more rigorous testing before wider spread.[35] Whatever analytical framework is adopted, Lamont and colleagues[36] have proposed five essential questions for evaluation which those planning to undertake evaluations of social prescribing programmes would do well to heed. These are:

Why—Clarify aims and establish what we already know from evidence
Who—Identify and engage stakeholders and likely users of research at outset
How—Think about study design, using an appropriate mix of methods, and adjust for bias where possible (or at least acknowledge)
What—Consider what to measure (activity, costs, outcomes) and combine data from different sources
When— Pay attention to timing of results to maximise impact

Alongside these, we would also emphasise that that rigorous conduct and transparent reporting (regardless of 'success' or 'failure') are essential. Reporting guidelines such as SQUIRE[37] with its focus on explaining 'Why did you start?', 'What did you do?', 'What did you find?' and 'What does it mean? could readily be applied to ensure that learning is

For peer review only - http://bmjopen.bmj.com/site/about/guidelines.xhtml

systematically captured in a generalisable format. This in turn would serve to ensure that any future decisions relating to the continuation or wider spread of social prescribing schemes are transparent and evidence informed.

Conclusions

Social prescribing is being widely advocated and implemented but current evidence fails to provide sufficient detail to judge either success or value for money. If social prescribing is to realise its potential, future evaluations must be comparative by design and consider when, for whom, how well and at what cost.

For peer review only

For peer review only - http://bmjopen.bmj.com/site/about/guidelines.xhtml

Contributors:

PMW took overall responsibility for the systematic review. LB, AB and PMW were involved in all stages of the review from development of the protocol, through screening studies and data extraction to analysis and synthesis and production of the final manuscript. KF provided input at all stages of the review and commented on drafts of the review. KW conducted literature searches and contributed to the methods section of the review. All authors approved the final version and PMW is the guarantor.

Funding:

This review was funded as part of research funded by the NIHR Health Services and Delivery Research programme (Project ref: 12/5002/18) with additional funding (for PMW) from the NIHR Collaboration for Leadership in Applied Health Research and Care (CLAHRC) Greater Manchester.

Disclaimer:

This report presents independent research funded by the National Institute for Health Research (NIHR). The views expressed are those of the authors and do not necessarily reflect those of the NIHR Health Services and Delivery Research programme, NIHR CLAHRC Greater Manchester or the Department of Health.

Data sharing

All available data can be obtained from the corresponding author.

For peer review only

References

1. NHS England. *Integrated Care Pioneer Programme: annual report 2014: pioneer profiles and case study examples*. Redditch: NHS England, 2014.

2. University of Westminster. *Report of the annual Social Prescribing Network conference*. London: University of Westminster, 2016.

3. Kimberlee R, Ward R, Jones M, et al. *Measuring the economic impact of Wellspring Healthy Living Centre's Social Prescribing Wellbeing Programme for low level mental health issues encountered by GP services*. University of the West of England, Bristol, 2014.

4. Department of Health, *White paper. Our health, our care, our say: a new direction for community services*. London: Crown Copyright, 2006.

5. Matthews-King A. *GP leader appointed clinical champion for social prescribing*. Pulse. London: Cogora Limited, 30 June 2016.

6. Committee. H. *Oral evidence: Public expenditure on health and social care*, HC 679 Q652, 2014.

7. Wilson PM, Farley K, Thompson C, et al. Effects of a demand-led evidence briefing service on the uptake and use of research evidence by commissioners of health services: protocol for a controlled before and after study. *Implement Sci* 2015;10:7.

8. Morton L, Ferguson M, Baty F. Improving wellbeing and self-efficacy by social prescription. *Pub Health* 2015; 129(3):286-9.

9. Jones M, Kimberlee R, Deave T, et al. The role of community centre-based arts, leisure and social activities in promoting adult well-being and healthy lifestyles. *Int J Environ Res Publ Health* 2013; 10(5):1948-62.

10. Orrow G, Kinmonth AL, Sanderson S, et al. Effectiveness of physical activity promotion based in primary care: systematic review and meta-analysis of randomised controlled trials. . *BMJ* 2012; 344:e1389

11. Pavey TG, Taylor AH, Fox KR, et al. Effect of exercise referral schemes in primary care on physical activity and improving health outcomes: systematic review and meta-analysis. *BMJ* 2011; 343:d6462:

12. Washburn RA, Lambourne K, Szabo AN, et al. Does increased prescribed exercise alter non-exercise physical activity/energy expenditure in healthy adults? A systematic review. *Clin Obes* 2013; 4:1-20.

13. Higgins JPT, Green S, (editors). *Cochrane Handbook for Systemtatic Reviews of Interventions Version 5.1.0* [updated March 2011]: The Cochrane Collaboration; 2011 [Available from: Available from www.cochrane-handbook.org].

14. National Heart LaBI. *Quality Assessment Tool for Before-After (Pre-Post) Studies With No Control Group*. 2014 [Available from:

For peer review only - http://bmjopen.bmj.com/site/about/guidelines.xhtml

https://www.nhlbi.nih.gov/health-pro/guidelines/in-develop/cardiovascular-risk-reduction/tools/before-after.

15. Kilgarriff-Foster A, Alicia OC. Exploring the components and impact of social prescribing. *J Public Ment Health* 2015;14(3):127-34.

16. Thomson LJ, Camic PM, Chatterjee HM. *Social Prescribing: A review of community referral schemes*. London: University College London, 2015.

17. Grant C, Goodenough T, Harvey I, et al. A randomized controlled trial and economic evaluation of a referrals facilitator between primary care and the voluntary sector. *BMJ* 2000; 320:419-23.

18. Maughan DL, Patel A, Parveen T, et al. Primary-care-based social prescribing for mental health: an analysis of financial and environmental sustainability. *Prim Health Care Res Dev* 2016; 17(2):114-21.

19. Dayson C BN. *The social and economic impact of the Rotherham Social Perscribing Pilot: Main Evaluation Report*. Centre for Regional Economic and Social Research, Sheffield Hallam University, 2014.

20. Friedli L, Themessl-Huber M, Butchart M. *Evaluation of Dundee Equally Well Sources of Support: Social Prescribing in Maryfield.* Evaluation Report Four, 2012.

21. Grayer J, Cape J, Orpwood L, et al. Facilitating access to voluntary and community services for patients with psychosocial problems: a before-after evaluation. *BMC Fam Pract* 2008; 9(27).

22. Faulkner M. Supporting the psychosocial needs of patients in general practice: the role of a voluntary referral service. *Patient Educ Couns* 2004; 52:41-46.

23. Age Concern Yorkshire and Humber. *Social Prescribing. A model for partnership working between primary care and the voluntary sector*. 2012.

24. Baines A. *Rugby Social Prescribing Project ConnectWELL. Harnessing community capacity to improve health and wellbeing: mid-term evaluation report*, 2015.

25. ERS Research and Consultancy. *Newcastle Social Prescribing Project: Final Report*. 2013.

26. Longwill A. *Independent evaluation of Hackney WellFamily service: Improving Health and Wellbeing*. 2014.

27. South J, Higgins TJ, Woodall J, et al. Can social prescribing provide the missing link? *Prim Health Care Res Dev* 2008; 9(04):310-18.

28. White J, Kinsella K, South J. *An evaluation of social prescribing health trainers in south and west Bradford*, 2010.

29. Woodall J, South J. *The evaluation of the CHAT social prescribing scheme in Bradford South and West PCT*, 2005.

30. Involve North East. *Newcastle West Clinical Commissioning Group social prescribing pilot*

For peer review only - http://bmjopen.bmj.com/site/about/guidelines.xhtml

project: the views of participating health professionals. 2013.

31. Brandling J, House W, Howitt D, et al. *New routes: pilot research project of a new social prescribing service provided in Keynsham*. 2011.

32. Cochrane Effective Practice and Organisation of Care Review Group (EPOC). *What study designs should be included in an EPOC review? EPOC Resources for review authors.* Oslo: Norwegian Knowledge Centre for the Health Services, 2013.

33. Auerbach AD, Landefeld CS, Shojania KG. The tension between needing to improve care and knowing how to do it. *N Engl J Med* 2007; 357(6):608-13.

34. Craig P, Dieppe P, Macintyre S, et al. Developing and evaluating complex interventions: the new Medical Research Council guidance. *BMJ* 2008;337:a1655.

35. Wilson PM, Boaden R, Harvey G. Plans to accelerate innovation in health systems are less than IDEAL. *BMJ Qual Saf* 2015; pii: bmjqs-2015-004605

36. Lamont T, Barber N, de Pury J, et al. New approaches to evaluating complex health and care systems. *BMJ* 2016;352:i154.

37. Ogrinc G, Davies L, Goodman D, et al. SQUIRE 2.0 (Standards for QUality Improvement Reporting Excellence): revised publication guidelines from a detailed consensus process. *BMJ Qual Saf* 2015; pii: bmjqs-2015-004411

For peer review only

For peer review only - http://bmjopen.bmj.com/site/about/guidelines.xhtml

Table 1: Characteristics of social prescribing project evaluations

**Project name, location**<br><br>**Author, year**	**Date project established (or time period of evaluation)**	**Referral activity**	**Participants in evaluation (excluding health professionals and link-workers)**	**Facilitator/Co-ordinator skills and training**	**Activities pa Social Prescr Facilitator/C**
Amalthea project, Avon<br><br>Grant, 2000	Aug 1997 to Sep 1998	**Referred to link-worker**: N=90<br><br>**Attended link-worker appointment:** 71/90 (79%)<br><br>**Attended a prescribed activity/services:** not reported<br><br>**GP surgeries involved:** N=26	**Approached to participate:** N=168<br><br>**Agreed to participate:** N=161<br>(90 randomised to intervention; 71 randomised to control)<br><br>**Included in evaluation analysis**: 69% of 90 for intervention an 67% of 71 for control followed up at 4 months	Three project facilitators from different backgrounds were trained and supervised by the organisation	Voluntary se<br>• Nationa<br>• Counsel<br>• Alcoholi<br>• Over Ea<br>• Local ea<br>• Triumph<br>• Woman<br>• Counsel<br>• CRUSE<br>• RELATE<br>• Befrienc<br>• Local ca<br>• Princess<br>• Royal Br<br>• Crisis<br>• Migrain<br>• Local as<br>• Nationa Prevent<br>• Multiple<br>• Disabilit<br>• British T Volunte<br>• Citizens<br>• Local m<br>• Local to<br>• Local so<br>• Universi<br>• Brunelc<br>• Battle a<br>• Women
Connect project, Carlisle<br><br>Maughan, 2016	Oct 2011 to Mar 2014	**Referred to link-worker**: not reported<br><br>**Attended link-worker appointment:** N=30<br><br>**Attended a prescribed activity/services:** not reported	**Approached to participate:** not reported<br><br>**Agreed to participate:** N=59<br>(30 in intervention group; 29 in control group)<br><br>**Included in evaluation analysis**: 28/30 (93%) in intervention; 29/29 (100%) in control	Non-healthcare staff, provided with brief training about local services, completing questionnaires and managing risk.<br><br>Not reported	Available se and private s self-manage educational, facilities and exercise-rela given: The E exchange an members ea another me community.

For peer review only

For peer review only - http://bmjopen.bmj.com/site/about/guidelines.xhtml

		**GP surgeries involved:** N=1			
Rotherham Social Prescribing project<br><br>Dayson, 2014	Apr 2012 to Mar 2014	**Referred to link-worker**: N=1607<br><br>**Attended link-worker appointment:** not reported<br><br>**Attended a prescribed activity/services:** not reported (1118 people were referred onwards to other funded voluntary and community sector services)<br><br>**GP surgeries involved:** N=29	**Approached to participate:** not reported<br><br>**Agreed to participate:** not reported<br><br>**Included in evaluation analysis**:<br>i. Hospital episode data analysis: N=451 followed up at 6 months;<br>N=108 followed at 12 months (of which n=42 referred<br>on to a funded voluntary and community service provider)<br>ii. Wellbeing outcomes analysis: 280/819 followed up at 3-4 months	Not reported.	Information activity; phy and enabling
Dundee Equally Well Sources of Support<br><br>Friedli, 2012	Mar 2011 to Jun 2012	**Referred to link-worker**: N=123<br><br>**Attended link-worker appointment:** 61/123 (50%)<br><br>**Attended a prescribed activity/services:** 26 out of 26 referred to an activity attended that activity (119 link-worker referrals were made into 47 different community services or groups)<br><br>**GP surgeries involved:** N=1	**Approached to participate:** not reported<br><br>**Agreed to participate:** not reported<br><br>**Included in evaluation analysis**: N=16	Not reported.	Community support and
Graduate Primary Care Mental Health Worker Community Link Scheme, north London	NR	**Referred to link-worker**: N=255<br><br>**Attended**	**Approached to participate:** N=151<br><br>**Agreed to participate:** 108/151	Psychology graduates with some voluntary clinical experience but no formal mental health training. In-house	Community through sear electronic di enquiries, ar

For peer review only - http://bmjopen.bmj.com/site/about/guidelines.xhtml

Grayer, 2008		**link-worker appointment:** N=151<br><br>**Attended a prescribed activity/services:** 58% attended at least one of the services suggested<br><br>**GP surgeries involved:** N=13	**Included in evaluation analysis**: N=75/108 followed up at 3 months	training and supervision from two clinical psychologists.<br><br>Not reported.	
Wellbeing Programme at Wellspring Healthy Living Centre, Bristol<br><br>Kimberlee, 2014	May 2012 to Apr 2013	**Referred to link-worker**: Unclear<br><br>**Attended link-worker appointment:** N=128<br><br>**Attended a prescribed activity/services:** not reported<br><br>**GP surgeries involved:** not reported	**Approached to participate:** N=128<br><br>**Agreed to participate:** N=128<br><br>**Included in evaluation analysis**:<br>i. Health and wellbeing outcomes N=70 followed up at 3 months<br>ii. GP attendance data N=40 12 months before and after baseline	Not reported	Peer suppor[illegible] physical acti[illegible] complement[illegible]
Age Concern, Yorkshire & Humber<br><br>Age Concern, 2012	Apr 2011 to Sep 2011	**Referred to link-worker**: N=55<br><br>**Attended link-worker appointment:** not reported<br><br>**Attended a prescribed activity/services:** not reported<br><br>**GP surgeries involved:** N=12	**Approached to participate:** unclear<br><br>**Agreed to participate:** unclear<br><br>**Included in evaluation analysis**: not reported	A skilled member of Age UK staff	Age UK servi[illegible] day clubs, lu[illegible] and advice, [illegible] theatre outi[illegible] advocacy, le[illegible] service, volu[illegible] classes, art g[illegible] services
ConnectWell, Coventry<br><br>Baines, 2015	Aug 2014 to Aug 2015	**Referred to link-worker**: N=39<br><br>**Attended link-worker appointment:** 24/39 (62%)	**Approached to participate:** not reported<br><br>**Agreed to participate:** not reported<br><br>**Included in evaluation analysis**: N=5	Volunteers attend group training session then inductions for specific role. Additional training offered e.g. mentoring, dementia awareness. Supervised by WCAVA	Befriending, information housing/hor[illegible] counselling, support grou[illegible]

For peer review only - http://bmjopen.bmj.com/site/about/guidelines.xhtml

		**Attended a prescribed activity/services:** not reported<br><br>**GP surgeries involved:** N=4			
Newcastle Social Prescribing Project<br><br>ERS Research and Consultancy, 2013<br><br>Involve North East, 2013	Jan 2012 to Mar 2013	**Referred to link-worker**: N=124<br><br>**Attended link-worker appointment:** 87/124 (70%)<br><br>**Attended a prescribed activity/services:** not reported<br><br>**GP surgeries involved:** N=6	**Approached to participate:** not reported<br><br>**Agreed to participate:** not reported<br><br>**Included in evaluation analysis**: N=9	Existing staff member in each VCSO with knowledge of local community and services, LTCs. Skills and attributes specified.	Support with and buddyin signposting t support thro<br>• Age UK<br>• HealthW<br>• Newcas<br>• Search<br>• West En
CHAT, south and west Bradford<br><br>Woodall, 2005	Established 2004 Piloted Jan 2005 to Sep 2005	**Referred to link-worker**: N=81<br><br>**Attended link-worker appointment:** not reported<br><br>**Attended a prescribed activity/services:** not reported<br><br>**GP surgeries involved:** N=3	**Approached to participate:** not reported<br><br>**Agreed to participate:** not reported<br><br>**Included in evaluation analysis**: N=10	Non-clinical Health Trainers, a public health workforce supported by the DH	Local commu services.
CHAT, south and west Bradford<br><br>South, 2008	May 2005 to Oct 2006	**Referred to link-worker**: N=223<br><br>**Attended link-worker appointment:** not reported<br><br>**Attended a prescribed activity/services:** not reported<br><br>**GP surgeries involved:** not reported	**Approached to participate:** not reported<br><br>**Agreed to participate:** not reported<br><br>**Included in evaluation analysis**: N=10	Non-clinical Health Trainers, a public health workforce supported by the DH	Community groups and s<br>• Lunche<br>• Befrienc<br>• Social se<br>• Volunte<br>• Getting<br>• Literacy<br>• Debt ad<br>• Access b<br>• Bereave<br>• Reminis<br>• Arts and<br>Music group

For peer review only

For peer review only - http://bmjopen.bmj.com/site/about/guidelines.xhtml

Health Trainer and Social Prescribing Service, south and west Bradford<br><br>White 2010	Established 2006 (evolved from CHAT) Jan 2010 to Sep 2010	**Referred to link-worker**: N=484<br><br>**Attended link-worker appointment:** not reported<br><br>**Attended a prescribed activity/services:** not reported<br><br>**GP surgeries involved:** N=21	**Approached to participate:** not reported<br><br>**Agreed to participate:** not reported<br><br>**Included in evaluation analysis**: N=12	Non-clinical Health Trainers, a public health workforce supported by the DH	Local volunta<br>social groups<br><br>Health traine<br>health action
Doncaster Patient Support Service<br><br>Faulkner, 2004	April 2001 to February 2002	**Referred to link-worker**: 200<br><br>**Attended link-worker appointment:** N=132<br><br>**Attended a prescribed activity/services:** Not reported<br><br>**GP surgeries involved:** N=1	**Approached to participate:** 17 patients and 9 volunteers<br><br>**Agreed to participate:** Patients: N=11 Volunteers: N=9<br><br>**Included in evaluation analysis**: Patients: N=11 Volunteers: N=9	Volunteers given 3 day training including basic counselling knowledge and skills, team building strategies, and visits from community services they might refer people to. Ongoing training and supervision provided.	Facilitated<br>providing: a<br>services, ac<br>alcohol sup<br>family/mat<br>support for<br>housing/so<br>(e.g. The W<br>Relate; Alco
WellFamily service in Hackney*<br><br>Longwill, 2014	First established 1996<br><br>Period of evaluation: 2012-13	**Referred to link-worker**: N=1466<br><br>**Attended link-worker appointment:** N=1089<br><br>**Attended a prescribed activity/services:** N=712<br><br>**GP surgeries involved:** 32	**Approached to participate:** Not reported<br><br>**Agreed to participate:** Not reported<br><br>**Included in evaluation analysis**: GAD7, PHQ9: N=387<br><br>Patient survey: N=92 respondents (out of active caseload of approx. 120)<br>GP survey: N=27 respondents (out of 160 surveyed GPs)	Family action workers and senior practitioners with a variety of skills and experience. Some with undergraduate and postgraduate qualifications in counselling, group therapy, medicine and psychotherapy.<br><br>Family Action counsellors - professionally qualified and under regular supervision	Short term c<br>practical sup<br>Local volunta<br>enterprise se<br><br>Other social<br>as debt coun<br>departments
'New Routes', Keynsham (Bath and North East Somerset)<br><br>Brandling, 2011	2-year pilot established October 2009	**Referred to link-worker**: N=90<br><br>**Attended link-worker appointment:** not	**Approached to participate:** Not reported<br><br>**Agreed to participate:** Not reported	Co-ordinators role modelled on Amalthea project[13]<br><br>Skills and training not reported	46 differen<br>and activiti<br>pilot.<br><br>Most popul<br>- volunte<br>- befrien

For peer review only - http://bmjopen.bmj.com/site/about/guidelines.xhtml

		reported<br><br>**Attended a prescribed activity/services:** N=42<br><br>**GP surgeries involved:** 3	**Included in evaluation analysis**:<br>WEMWBS completed at 6-12 months N=7<br>MYMOP2 completed at 6-12 months N=12<br><br>Qualitative interviews N=21		- walking<br>- art grou[illegible]

NR, not reported; WEMWBS, Warwick Edinburgh Mental Wellbeing Scale; MYMOP2, Measure Yourself Medical Outcome Profile

For peer review only

For peer review only - http://bmjopen.bmj.com/site/about/guidelines.xhtml

Table 2: Quality assessment and risk of bias

Comparative evaluations

Study	Quality criteria	Risk of bias	Notes
**Grant 2000**<br><br>**RCT**	Sequence generation	Low	Sealed opaque envelopes prepared by research team. Stratification by practice and blocks of six used (3 intervention/3 control).
	Allocation concealment	Unclear	Sequentially numbered envelopes opened. In two practices there was evidence that the randomization process was initially misunderstood: six patients excluded.
	Blinding of participants and personal	Not possible	
	Blinding of outcome assessment	Unclear	
	Incomplete outcome data	High	32% loss to follow-up at 4 months
	Selective outcome reporting	Unclear	
	Other potential threats to validity	Unclear	Numbers potentially eligible but not recruited unknown<br>Recruited general practices were not a random sample: participating doctors were likely to be more interested in the research question and may have managed psychosocial problems more actively, which could have diminished reported estimates of effects
**Maughan 2016**<br><br>**CBA**	Is there a suitable comparison group?	Yes	One intervention and one control group, drawn from the same general practice with similar patient characteristics.<br>Models environmental costs (in terms of carbon footprint)<br>Data were retrospectively collected from GP health records for a two-year period.<br>Two participants in intervention group excluded from analysis<br>Financial and environmental impacts calculated for each outcome using national averages or accepted conversion factors
	Do the authors use theory to underpin the project/evaluation?	No	
	Were appropriate methods used for data collection and analysis?	Yes	
	Were efforts made to assess patient experience?	No	

Uncontrolled before and after evaluations

Study	Quality criteria	Judgement	Notes
**Dayson 2014**	Was the study question or objective clearly stated?	Yes	Small sample of those referred (N=1607) participated in evaluation – HES data at months N=451, at 12 months N=10; wellbeing data at 3-4 months 280/819<br><br>Methods of qualitative analysis of patient experience unclear
	Were eligibility/selection criteria for the study population prespecified and clearly described?	Not reported	
	Were the participants in the study representative of those who would be eligible for the test/service/intervention in the	Yes	

For peer review only

	general or clinical population of interest?		
	Were all eligible participants that met the prespecified entry criteria enrolled?	Not reported	
	Was the sample size sufficiently large to provide confidence in the findings?	No	
	Was the test/service/intervention clearly described and delivered consistently across the study population?	Not reported	
	Were the outcome measures prespecified, clearly defined, valid, reliable, and assessed consistently across all study participants?	Yes	
	Were the people assessing the outcomes blinded to the participants' exposures/interventions?	Not reported	
	Was the loss to follow-up after baseline 20% or less? Were those lost to follow-up accounted for in the analysis?	No	
	Did the statistical methods examine changes in outcome measures from before to after the intervention? Were statistical tests done that provided p values for the pre-to-post changes?	Yes	
	Were outcome measures of interest taken multiple times before the intervention and multiple times after the intervention (i.e., did they use an interrupted time-series design)?	No	
	If the intervention was conducted at a group level (e.g., a whole hospital, a community, etc.) did the statistical analysis take into account the use of individual-level data to determine effects at the group level?	Not applicable	
**Friedli 2012**	Was the study question or objective clearly stated?	Yes	Details of pre and post intervention outcomes not reported<br>Small sample size<br>Timing of post intervention assessment not reported<br>Methods of qualitative analysis of patient and provider/referrer experience unclear
	Were eligibility/selection criteria for the study population prespecified and clearly described?	No	
	Were the participants in the study representative of those who would be eligible for the test/service/intervention in the general or clinical population of interest?	Yes	
	Were all eligible participants that met the prespecified entry criteria enrolled?	Not applicable	
	Was the sample size sufficiently	No	

For peer review only - http://bmjopen.bmj.com/site/about/guidelines.xhtml

	large to provide confidence in the findings?		
	Was the test/service/intervention clearly described and delivered consistently across the study population?	Not reported	
	Were the outcome measures prespecified, clearly defined, valid, reliable, and assessed consistently across all study participants?	No	
	Were the people assessing the outcomes blinded to the participants' exposures/interventions?	No	
	Was the loss to follow-up after baseline 20% or less? Were those lost to follow-up accounted for in the analysis?	No	
	Did the statistical methods examine changes in outcome measures from before to after the intervention? Were statistical tests done that provided p values for the pre-to-post changes?	No	
	Were outcome measures of interest taken multiple times before the intervention and multiple times after the intervention (i.e., did they use an interrupted time-series design)?	No	
	If the intervention was conducted at a group level (e.g., a whole hospital, a community, etc.) did the statistical analysis take into account the use of individual-level data to determine effects at the group level?	Not applicable	
**Grayer 2008**	Was the study question or objective clearly stated?	Yes	GP practices volunteered and may not be representative of practices overall<br>Patients who consented to participate in evaluation were more likely to speak English as a first language than those who did not consent<br>No significant differences at baseline between those successfully followed up and those lost to follow up<br>95% confidence intervals (no P values) reported for changes in GHQ-12, CORE-OM and WSAS scores
	Were eligibility/selection criteria for the study population prespecified and clearly described?	Yes	
	Were the participants in the study representative of those who would be eligible for the test/service/intervention in the general or clinical population of interest?	Yes	
	Were all eligible participants that met the prespecified entry criteria enrolled?	No	
	Was the sample size sufficiently large to provide confidence in the findings?	No	
	Was the test/service/intervention clearly described and delivered consistently across the study population?	Yes	

For peer review only - http://bmjopen.bmj.com/site/about/guidelines.xhtml

	Were the outcome measures prespecified, clearly defined, valid, reliable, and assessed consistently across all study participants?	Yes	
	Were the people assessing the outcomes blinded to the participants' exposures/interventions?	Not reported	
	Was the loss to follow-up after baseline 20% or less? Were those lost to follow-up accounted for in the analysis?	No	
	Did the statistical methods examine changes in outcome measures from before to after the intervention? Were statistical tests done that provided p values for the pre-to-post changes?	Yes	
	Were outcome measures of interest taken multiple times before the intervention and multiple times after the intervention (i.e., did they use an interrupted time-series design)?	No	
	If the intervention was conducted at a group level (e.g., a whole hospital, a community, etc.) did the statistical analysis take into account the use of individual-level data to determine effects at the group level?	Not applicable	
**Kimberlee 2014**	Was the study question or objective clearly stated?	Yes	SROI analysis presents data for all baseline completers and the smaller percentage who were followed up; possible bias towards positive finding for intervention<br><br>Unclear whether calculations of mean differences in scale scores used all baseline data or baseline data for follow up completers only<br><br>P values reported for change from baseline at 3 months in PHQ-9 depression scores
	Were eligibility/selection criteria for the study population prespecified and clearly described?	No	
	Were the participants in the study representative of those who would be eligible for the test/service/intervention in the general or clinical population of interest?	Yes	
	Were all eligible participants that met the prespecified entry criteria enrolled?	Not applicable	
	Was the sample size sufficiently large to provide confidence in the findings?	No	
	Was the test/service/intervention clearly described and delivered consistently across the study population?	Not reported	
	Were the outcome measures prespecified, clearly defined, valid, reliable, and assessed consistently across all study participants?	Yes	
	Were the people assessing the outcomes blinded to the	Not reported	

For peer review only - http://bmjopen.bmj.com/site/about/guidelines.xhtml

	participants' exposures/interventions?		
	Was the loss to follow-up after baseline 20% or less? Were those lost to follow-up accounted for in the analysis?	No	
	Did the statistical methods examine changes in outcome measures from before to after the intervention? Were statistical tests done that provided p values for the pre-to-post changes?	Yes	
	Were outcome measures of interest taken multiple times before the intervention and multiple times after the intervention (i.e., did they use an interrupted time-series design)?	No	
	If the intervention was conducted at a group level (e.g., a whole hospital, a community, etc.) did the statistical analysis take into account the use of individual-level data to determine effects at the group level?	Not applicable	

For peer review only

Table 3: Health and wellbeing outcomes (validated measures)

Study (timing of outcome measurement post baseline measurement)	WEMWBS	HADS	GAD-7	PHQ-9	CORE-OM	WSAS
**RCTs**						
**Grant 2000 (4 months)**		Intervention group (N=62)* greater improvement than control group (N=48)*				
**Before and after evaluations**						
**Friedli 2012 (NR)**	"Statistically significant improvement" in mental wellbeing (N=16) (scores not reported)					"Statistically significant improvement" in functional ability (N=16)(scores not reported)
**Grayer 2008 (3 months)**					Small reduction in patients categorised as cases (N=74)	Improvement in work and social adjustment (N=69)
**Descriptive reports**						
**ERS Research and Consultancy 2013 (NR)**	Increase in mean score from 22 to 26 (N=16)					
**Longwill 2014 (NR)**			2.5 point reduction in score (P<0.001) (N=387)	3.1 point reduction in score (P<0.001) (N=387)		
**Brandling 2011 (6-12 months)**	"General positive trend but owing to low number of participants completing questionnaires no further conclusions can be made"					

*calculated from reported percentage followed up at 4 months

WEMWBS: Warwick-Edinburgh Mental Wellbeing Scale; HADS: Hospital Anxiety and Depression Scale; GAD-7: General Anxiety Disorder-7; PHQ-9: Patient Health Questionnaire-9; CORE-OM: Core

For peer review only - http://bmjopen.bmj.com/site/about/guidelines.xhtml

Outcome Measure; WSAS: Work and Social Adjustment Scale; GHQ-12: General Health Questionnaire-12; COOP/WONCA: Dartmouth COOP Functional Health Assessment Charts

For peer review only

For peer review only - http://bmjopen.bmj.com/site/about/guidelines.xhtml

Figure 1: PRISMA Flow Diagram

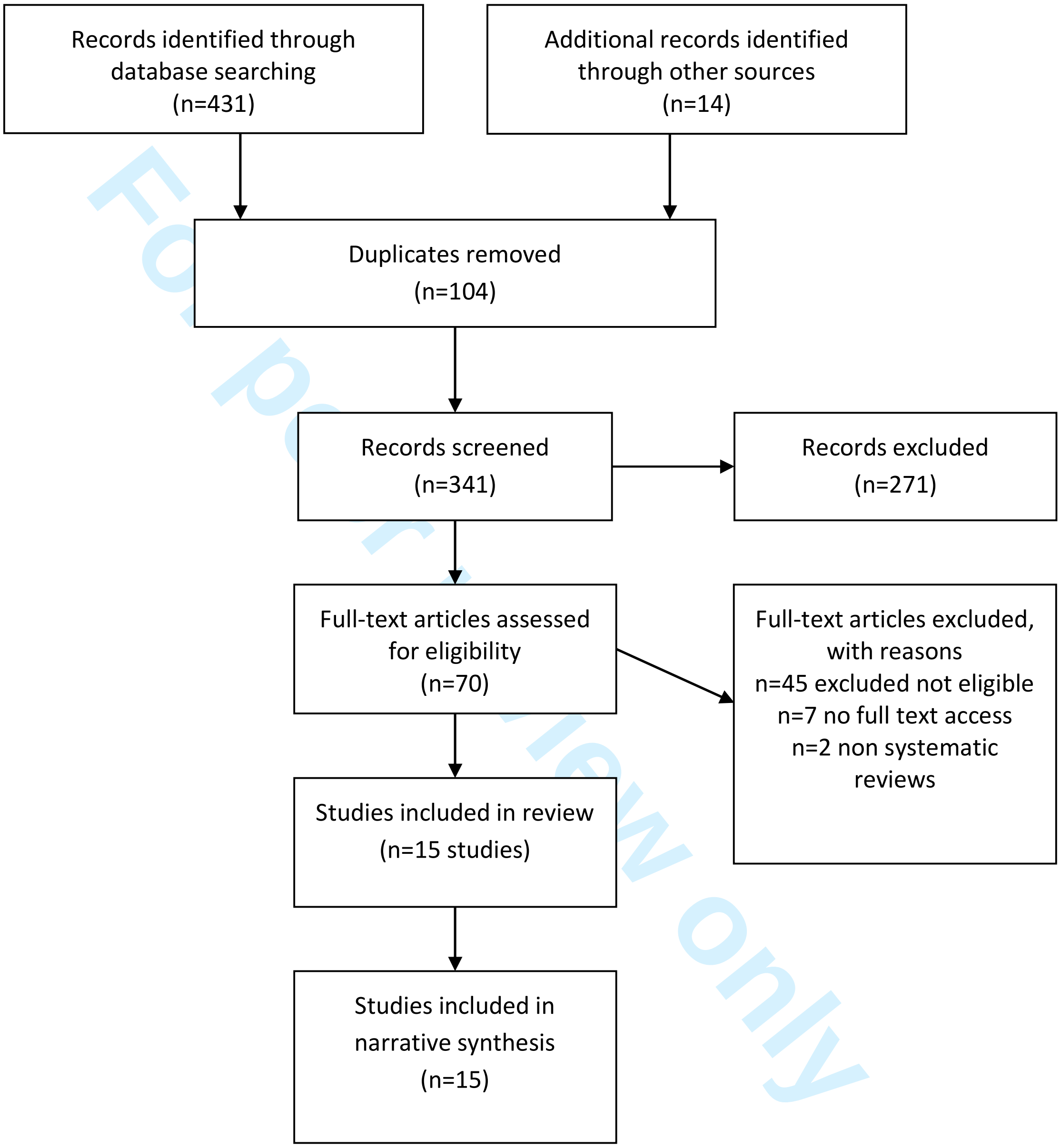

For peer review only - http://bmjopen.bmj.com/site/about/guidelines.xhtml

Appendix 1: Search strategies

ASSIA via Proquest Search date 26th June 2015 and 5th February 2016

"social prescrib*" OR "social prescrip*" OR "community referral*"

CINAHL via EBSCO search date 26th June 2015 and 5th February 2016

social prescribing OR "social prescrip*" OR "community referral*"

Ovid MEDLINE(R) In-Process & Other Non-Indexed Citations and Ovid MEDLINE(R) <1946 to Present> searched 26th June 2015 and 5th February 2016

1 social prescrib$.ti,ab.
2 social prescrip$.ti,ab.
3 community referral$.ti,ab.
4 non-medical referral$.ti,ab.
5 well being program$.ti,ab.
6 well-being program$.ti,ab.
7 wellbeing program$.ti,ab.
8 1 or 2 or 3 or 4 or 5 or 6 or 7

Social Care Online via http://www.scie-socialcareonline.org.uk/ searched 26th June 2015 and 5th February 2016

"Social prescribing" OR "social prescription*" or "community referral*"

Social Policy & Practice via OVID search date 26th June 2015 and 5th February 2016

1 social prescrib$.ti,ab.
2 social prescrip$.ti,ab.
3 community referral$.ti,ab.
4 non-medical referral$.ti,ab.
5 well being program$.ti,ab.
6 well-being program$.ti,ab.
7 wellbeing program$.ti,ab.
8 1 or 2 or 3 or 4 or 5 or 6 or 7

Google search last performed 5th January 2016

Two reviewers independently searched google.co.uk using the search terms "social prescribing" and "community referral" and reviewed the search results from the first 10 pages

For peer review only - http://bmjopen.bmj.com/site/about/guidelines.xhtml

PRISMA 2009 Checklist

Section/topic	#	Checklist item	Reported on page #
**TITLE**			
Title	1	Identify the report as a systematic review, meta-analysis, or both.	1
**ABSTRACT**			
Structured summary	2	Provide a structured summary including, as applicable: background; objectives; data sources; study eligibility criteria, participants, and interventions; study appraisal and synthesis methods; results; limitations; conclusions and implications of key findings; systematic review registration number.	2
**INTRODUCTION**			
Rationale	3	Describe the rationale for the review in the context of what is already known.	4
Objectives	4	Provide an explicit statement of questions being addressed with reference to participants, interventions, comparisons, outcomes, and study design (PICOS).	4
**METHODS**			
Protocol and registration	5	Indicate if a review protocol exists, if and where it can be accessed (e.g., Web address), and, if available, provide registration information including registration number.	2
Eligibility criteria	6	Specify study characteristics (e.g., PICOS, length of follow-up) and report characteristics (e.g., years considered, language, publication status) used as criteria for eligibility, giving rationale.	5
Information sources	7	Describe all information sources (e.g., databases with dates of coverage, contact with study authors to identify additional studies) in the search and date last searched.	5
Search	8	Present full electronic search strategy for at least one database, including any limits used, such that it could be repeated.	Supp File
Study selection	9	State the process for selecting studies (i.e., screening, eligibility, included in systematic review, and, if applicable, included in the meta-analysis).	5-6
Data collection process	10	Describe method of data extraction from reports (e.g., piloted forms, independently, in duplicate) and any processes for obtaining and confirming data from investigators.	6
Data items	11	List and define all variables for which data were sought (e.g., PICOS, funding sources) and any assumptions and simplifications made.	5-6
Risk of bias in individual studies	12	Describe methods used for assessing risk of bias of individual studies (including specification of whether this was done at the study or outcome level), and how this information is to be used in any data synthesis.	6
Summary measures	13	State the principal summary measures (e.g., risk ratio, difference in means).	n/a
Synthesis of results	14	Describe the methods of handling data and combining results of studies, if done, including measures of consistency (e.g., I^2) for each meta-analysis.	n/a

For peer review only - http://bmjopen.bmj.com/site/about/guidelines.xhtml

PRISMA 2009 Checklist

Section/topic	#	Checklist item	Reported on page #
Risk of bias across studies	15	Specify any assessment of risk of bias that may affect the cumulative evidence (e.g., publication bias, selective reporting within studies).	n/a
Additional analyses	16	Describe methods of additional analyses (e.g., sensitivity or subgroup analyses, meta-regression), if done, indicating which were pre-specified.	n/a
**RESULTS**			
Study selection	17	Give numbers of studies screened, assessed for eligibility, and included in the review, with reasons for exclusions at each stage, ideally with a flow diagram.	7
Study characteristics	18	For each study, present characteristics for which data were extracted (e.g., study size, PICOS, follow-up period) and provide the citations.	17-23
Risk of bias within studies	19	Present data on risk of bias of each study and, if available, any outcome level assessment (see item 12).	8
Results of individual studies	20	For all outcomes considered (benefits or harms), present, for each study: (a) simple summary data for each intervention group (b) effect estimates and confidence intervals, ideally with a forest plot.	29
Synthesis of results	21	Present results of each meta-analysis done, including confidence intervals and measures of consistency.	n/a
Risk of bias across studies	22	Present results of any assessment of risk of bias across studies (see Item 15).	8
Additional analysis	23	Give results of additional analyses, if done (e.g., sensitivity or subgroup analyses, meta-regression [see Item 16]).	n/a
**DISCUSSION**			
Summary of evidence	24	Summarize the main findings including the strength of evidence for each main outcome; consider their relevance to key groups (e.g., healthcare providers, users, and policy makers).	11
Limitations	25	Discuss limitations at study and outcome level (e.g., risk of bias), and at review-level (e.g., incomplete retrieval of identified research, reporting bias).	11
Conclusions	26	Provide a general interpretation of the results in the context of other evidence, and implications for future research.	13
**FUNDING**			
Funding	27	Describe sources of funding for the systematic review and other support (e.g., supply of data); role of funders for the systematic review.	14

From: Moher D, Liberati A, Tetzlaff J, Altman DG, The PRISMA Group (2009). Preferred Reporting Items for Systematic Reviews and Meta-Analyses: The PRISMA Statement. PLoS Med 6(7): e1000097. doi:10.1371/journal.pmed1000097

For more information, visit: **www.prisma-statement.org**.

For peer review only - http://bmjopen.bmj.com/site/about/guidelines.xhtml

BMJ Open

Social prescribing: less rhetoric and more reality. A systematic review of the evidence

Journal:	*BMJ Open*
Manuscript ID	bmjopen-2016-013384.R1
Article Type:	Research
Date Submitted by the Author:	15-Nov-2016
Complete List of Authors:	Bickerdike, Liz; University of York, Centre for Reviews and Dissemination<br>Booth, Alison; University of York, Dept of Health Sciences<br>Wilson, Paul; University of Manchester, Alliance Manchester Business School<br>Farley, Kate; University of Leeds, School of Healthcare<br>Wright, Kath; University of York, Centre for Reviews & Dissemination
**Primary Subject Heading**:	Health services research
Secondary Subject Heading:	General practice / Family practice
Keywords:	Health policy < HEALTH SERVICES ADMINISTRATION & MANAGEMENT, Organisation of health services < HEALTH SERVICES ADMINISTRATION & MANAGEMENT, PRIMARY CARE

SCHOLARONE™ Manuscripts

For peer review only

Social prescribing: less rhetoric and more reality. A systematic review of the evidence

Liz Bickerdike[1], Alison Booth[2], Paul M Wilson[3], Kate Farley[4], Kath Wright[1]

1. Centre for Reviews and Dissemination, University of York
2. York Trials Unit, University of York
3. Alliance Manchester Business School, University of Manchester
4. School of Healthcare, University of Leeds

Address for correspondence

Paul Wilson
Alliance Manchester Business School
University of Manchester
Booth Street East
Manchester
M15 6PB

Email: paul.wilson@manchester.ac.uk

For peer review only

For peer review only - http://bmjopen.bmj.com/site/about/guidelines.xhtml

Objectives: Social prescribing is a way of linking patients in primary care with sources of support within the community to help improve their health and well-being. Social prescribing programmes are being widely promoted and adopted in the UK NHS and so we conducted a systematic review to assess the evidence for their effectiveness.

Setting/data sources: Nine databases were searched from 2000 to January 2016 for studies conducted in the UK. Relevant reports and guidelines, websites and reference lists of retrieved articles were scanned to identify additional studies. All the searches were restricted to English language only.

Participants: Systematic reviews and any published evaluation of programmes where patient referral was made from a primary care setting to a link-worker or facilitator of social prescribing were eligible for inclusion. Risk of bias for included studies was undertaken independently by two reviewers and a narrative synthesis was performed.

Primary and secondary outcome measures: Primary outcomes of interest were any measures of health and wellbeing and or utilisation of health services.

Results: We included a total of 15 evaluations of social prescribing programmes. Most were small scale and limited by poor design and reporting. All were rated as a having a high risk of bias. Common design issues included a lack of comparative controls, short follow up durations, a lack of standardised and validated measuring tools, missing data and a failure to consider potential confounding factors. Despite clear methodological shortcomings, most evaluations presented positive conclusions.

Conclusions: Social prescribing is being widely advocated and implemented but current evidence fails to provide sufficient detail to judge either success or value for money. If social prescribing is to realise its potential, future evaluations must be comparative by design and consider when, by whom, for whom, how well and at what cost.

Trial registration: PROSPERO Registration: CRD42015023501

For peer review only - http://bmjopen.bmj.com/site/about/guidelines.xhtml

Strengths and limitations

Social prescribing is a way of linking patients in primary care with sources of support within the community. It is being widely promoted and adopted as means of dealing with some of the pressures on general practice.

This systematic review assesses the effectiveness of social prescribing programmes relevant to the UK NHS setting. We have searched for full publications and grey literature since 2000 and identified 15 evaluations. It is possible that some local evaluations have not been identified but it is unlikely that any unidentified evaluations would do little to alter the overall picture of a low quality evidence base with a high risk of bias.

If social prescribing is to realise its potential, future evaluations must be comparative by design and consider when, for whom, how well and at what cost.

For peer review only

For peer review only - http://bmjopen.bmj.com/site/about/guidelines.xhtml

Background

With estimates of a £30 billion funding gap by 2020, a radical rethink of the way health services are currently delivered remains high on the policy agenda. The Five Year Forward View has stressed that developing innovative approaches to delivering health care are integral to the long term future of the National Health Service (NHS).[1]

Social prescribing is one such model and is being widely promoted as a way of making general practice more sustainable. Social prescribing is a way of linking patients in primary care with sources of support within the community. It provides GPs with a non-medical referral option that can operate alongside existing treatments to improve health and well-being. There is no widely agreed definition of social prescribing but the Social Prescribing Network defines it as 'enabling healthcare professionals to refer patients to a link worker, to co-design a nonclinical social prescription to improve their health and wellbeing.'[2] Schemes commonly utilise services provided by the voluntary and community sector and can include an extensive range of practical information and advice, community activity, physical activities, befriending and enabling services. The types of activities offered as part of a social prescribing service can aim to help address the psychological problems and low levels of wellbeing often manifest in frequent attenders in general practice. By addressing these it is often hoped that there will be a subsequent positive impact on frequency of attendance.[3]

As early as 1999, the white paper *Saving Lives: Our Healthier Nation* was advocating that the NHS should make better use of community support structures and voluntary organisations.[4] However, it was in 2006 that the Department of Health advocated the introduction of social prescriptions for those with long-term conditions,[5] and NHS England have since announced the appointment of a national clinical champion for social prescribing.[6] With the current Secretary of State for Health also promoting access to non-clinical interventions that take a more 'holistic view',[1 7] support for social prescribing is significant at the policy level.

Many localities are now offering or considering implementing social prescribing programmes, but is the apparent enthusiasm justified? As part of a study which aimed to help NHS commissioners make better use of research in their decision making,[8] we examined the evidence for social prescribing. This systematic review summarises the evidence for the effectiveness of social prescribing programmes relevant to the UK NHS setting.

For peer review only - http://bmjopen.bmj.com/site/about/guidelines.xhtml

Methods

The protocol and amendments were registered in PROSPERO (Registration number: CRD42015023501).

Data sources and searches

DARE, Cochrane Database of Systematic Reviews and NHS EED were searched for relevant systematic reviews and economic evaluations (24th June 2015; no new records added to DARE and NHS EED databases from January 2015 so we did not run updated searches).

We searched the following databases (initial search 26th June 2015; updated search 5th February 2016): ASSIA, CINAHL, MEDLINE, Social Care Online and Social Policy & Practice.

As our focus was on identifying evidence relevant to the UK NHS setting we also searched for eligible studies in key UK knowledge repositories for health and social care. The websites of NICE, SCIE and NHS Evidence were searched for reviews, guidance, evidence briefings or any other papers describing or evaluating social prescribing programmes. Additional searches of the websites of key policy think tanks the Kings Fund, Health Foundation, Nuffield Trust and NESTA were also undertaken. We searched Google to identify grey literature reports of relevant evaluations in UK settings (5th January 2016). Reference lists of retrieved articles were scanned to identify additional studies.

All the searches were restricted to English language only and published between 2000 to January 2016. The search strategies are available in Appendix 1.

Study selection

Systematic reviews and any published evaluation of programmes where healthcare professionals refer patients from a primary care setting to a link-worker or facilitator for any form of social prescription were eligible for inclusion. Studies were eligible regardless of whether a comparison group was included.

As per the Social Prescribing Network definition, we included only studies where referral was made from a primary care setting to a co-ordinator, link-worker or facilitator of social prescribing (this type of role will be referred to as "link-workers" throughout this review). Any activities or interventions being specifically delivered as part of a social prescribing programme were included in the review.

We excluded studies where referral was made from outside of a primary care setting[9] and or

For peer review only - http://bmjopen.bmj.com/site/about/guidelines.xhtml

For peer review only

where primary care health professional refer patients to services delivered as part of mental health or counselling services such as an Improving Access to Psychological Therapies (IAPT) programme. We also excluded evaluations of activities that could be socially prescribed (for example physical activity programmes or community arts projects) but did not involve referral to a link-worker in the first instance. [10-13]

The primary outcomes of interest were any measures of health and wellbeing, including self-reported measures (for example levels of physical activity or depression scores) and or measure of utilisation of health services. We also considered any other outcomes (e.g. health service utilisation) reported in the included evaluations.

Study selection was performed by one researcher and checked by a second, with any discrepancies resolved by discussion or with recourse to a third researcher.

Data extraction and quality assessment

Details of the setting, participants, the intervention (type, delivery mode and length of time), type of evaluation and outcomes of evaluation were extracted and quality assessed by one researcher and checked by a second. Discrepancies were resolved by discussion or by recourse to a third researcher.

We used the Cochrane risk of bias tool to assess the quality of the randomised controlled trial.[14] To assess the quality of the before and after evaluations we applied the quality assessment tool developed by the US National Heart, Lung and Blood Institute for before-after (pre-post) studies with no control group.[15] Our primary focus was on effects. As per our protocol, we have not made a formal quality assessment of studies of a qualitative or descriptive nature.

Data synthesis and analysis

We performed a narrative synthesis of the evidence. There was insufficient data to perform meta-analysis for any of the outcomes of interest. No subgroup analyses were planned. The narrative synthesis was intended to move beyond a preliminary summary of study findings and quality to investigate similarities and differences between studies as well as exploring any patterns in the data.

For peer review only - http://bmjopen.bmj.com/site/about/guidelines.xhtml

Results

We identified a total of 431 records through database searching and a further 14 records through other sources. After deduplication 341 titles and abstracts were screened and 70 full text papers were assessed for inclusion (see Figure 1: PRISMA flow diagram).

Excluded studies

We excluded 45 studies on eligibility grounds and were unable to access the full text for seven identified records. We also identified two non-systematic reviews of social prescribing schemes.[16 17] These were excluded as they did not critically appraise included studies and were limited in their synthesis of findings; one review included a number of evaluations that did not meet our inclusion criteria.[17] We checked the reference lists of both reviews to ensure we had identified and included all relevant evaluations.

Included studies

We included a total of 15 evaluations (reported in 16 papers) of social prescribing programmes where some form of link-worker role was utilised.[3 18-32] The designs included one RCT,[18] one non-RCT,[19] two qualitative studies,[23 28] four uncontrolled before and after studies,[3 20-22] and eight descriptive reports of six evaluations, of which five included some analysis of qualitative data.[24-27 29-32] Details of the included evaluations are presented in Table 1.

In each of the included studies, the link-worker (job title variously named) met with the patient to discuss their needs and directed them to appropriate community/voluntary sector sources of support in their locality. The training and knowledge of people fulfilling these types of link-worker role varied between projects. In some services this was a paid role, in others these roles were fulfilled by volunteers. Some link-workers had good knowledge and existing networks with local services in place [28-30] and in others they received some basic training and made use of a directory of resources.[22]

Patients were referred to a range of activities provided by local or national voluntary and community sector organisations. Interventions received included exercise and other physical activities, signposting to housing, welfare and debt advice, adult education and literacy, befriending, counselling, self-help support groups, luncheon clubs and art activities.

The number of referrals made to social prescribing programmes ranged from 30 to 1607. Referrals were made by a range of health professionals but primarily GPs. Three of the studies reported that feedback was given to the referrer about the actions taken and the

For peer review only - http://bmjopen.bmj.com/site/about/guidelines.xhtml

participants' progress in the social prescribing programme.[22 28 30]

Quality of the evidence

Quality assessment and risk of bias for the evaluative designs is presented in Table 2. In the randomised controlled trial only sequence generation was adjudged to be of low risk of bias; all other criteria were rated as unclear or high risk.[18] The authors reported that the randomisation process was misunderstood in two of the participating practices but random allocation appeared to be maintained. A key inclusion criteria for the Cochrane Effective Practice and Organisation of Care Review Group is that a controlled before and after study must have at least two intervention and two control groups to guard against confounding.[33] Here, the controlled before and after study includes one intervention and one control group, drawn from the same general practice. As such, we rated the study as having a high risk of bias and made no further assessment of quality with the Cochrane risk of bias tool. Uncontrolled before-and-after studies are inherently weak evaluative designs and no included study fulfilled all of the specified quality criteria. In general, evaluations had small sample sizes (less than 100 participants), significant loss to follow up (>20%), were lacking in completeness of outcome data and had unclear selection criteria for the study population. Follow-up periods were generally short (immediately post-intervention up to 4 months post-intervention). There is a therefore a high risk of bias.

Uptake and attendance

Seven included studies reported the number of people attending an initial appointment with a link-worker. Where reported, attendance at this initial appointment with a link-worker ranged from 50% to 79%.[18 21-23 25-27] Participants' attendance at activities to which they were subsequently referred or recommended by a link-worker was reported in only two studies and varied from 58% [22] to 100%.[21]

Health and wellbeing outcomes

The RCT [18], two uncontrolled before-and-after studies [21 22] and three descriptive reports [26 27 32] measured health and wellbeing outcomes at baseline and again at up to 6 months after referral to a social prescribing programme; one study reported outcomes at up 12 months. The measures used were Warwick-Edinburgh Mental Wellbeing Scale (WEMWBS;[21 26 32]), Hospital Anxiety and Depression Scale (HADS;[18]), General Anxiety Disorder-7 (GAD-7;[27]); Patient Health Questionnaire-9 (PHQ-9):[27]); Clinical Outcomes in Routine Evaluation-Outcome Measure (CORE-OM);[22], WSAS ([21 22]), General Health Questionnaire (GHQ-12; [22]) and COOP/WONCA.[18] Table 3 presents findings for studies using validated measures; all report

For peer review only - http://bmjopen.bmj.com/site/about/guidelines.xhtml

some improvements in health and wellbeing. However it is difficult to quantify the size of the observed improvements due to a lack of reported detail, a lack of sufficient control group data, and differences in reporting between studies. It is not possible to determine whether any observed improvements were clinically significant. Studies reported short-term outcomes only; there is no evidence about the effect social prescribing has on health and wellbeing outcomes beyond six months.

One uncontrolled before and after study used a bespoke measure, the Wellspring Wellbeing Questionnaire, comprising PHQ9 and GAD7 tools, and items from ONS's Wellbeing Index/Integrated Household Survey and International Physical Activity Questionnaires.[3] A second also used a bespoke measure which utilised a 5-point scale across eight domains associated with different aspects of self-management such as 'looking after yourself' and 'managing symptoms'.[20] Two further descriptive reports also indicated they used the WEMWBS to measure changes in health and wellbeing but poor reporting and what appears to be very small numbers of responders.[24 25] In the two studies using non-validated measures some positive improvements in outcomes such as depression and anxiety at 3 to 4 months' follow up were reported.[3 20]

Health care utilisation outcomes

Both comparative evaluations[18 19] and three uncontrolled before and after studies[3 20 22] reported some measure of health care utilisation. This included comparing hospital episode statistics (HES) and/or GP record data from 6 to 12 months before intervention with data up to 18 months post intervention. Reported outcomes included frequency of GP consultations, referrals to secondary care, in-patient admissions and A&E attendances. Findings were mixed. The RCT reported that the number of primary care contacts were similar between intervention and control groups and that there were fewer referrals to secondary care and more prescription drugs for those in the intervention group compared with the control group.[18] The non-randomised trial reported statistically non-significant reductions in primary care contacts (face-to-face and/or telephone) and referrals to secondary care[19]. The before and after studies reported reductions in secondary care referrals, in-patient admissions and A&E attendances,[20] in primary care contact,[22] in face-to-face GP contact but an increase in telephone contact.[3]

Patient experience

Three before and after studies[20-22] and five descriptive reports[23 26 28 30 32] reported on patient experience. Studies used semi-structured interviews or survey questionnaires specifically

For peer review only - http://bmjopen.bmj.com/site/about/guidelines.xhtml

designed for the project evaluation to assess participant experience.

In six of the studies, participants reported overall satisfaction with social prescribing programmes.[20-22 26 28 30] General improvements in feelings of loneliness and social isolation,[21 30 32] and improved mental and physical health were also observed.[21] Issues that may impact the willingness of patients to participate in socially prescribed activities included confidence,[21 30] interest in/appropriateness of activities on offer[21 30] and literacy or travel issues.[30 32] One qualitative study reported that patients had poor knowledge of the service prior to attending their appointment with the link-worker resulting in some feeling that the service did not meet their expectations.[23] Another evaluation identified a similar issue regarding a lack of understanding of the service among participants.[32]

Referrer experience and lessons learned

A small number of studies conducted semi-structured interviews with primary care practitioners referring participants to social prescribing programmes and/or link-workers.[21 26 28-32] GPs in general found that being able to make a social prescription was a useful additional tool.[21 28 29 31] Key issues identified for successful implementation of social prescribing programmes were central coordination of referrals,[26] resources and training to support co-ordinators and enabling networking with the voluntary and community sector,[26 29] and good communication between GPs, participants and link-workers: social prescribing is unfamiliar to many GPs and requires good clear explanation to engage participants[21 23 26 32]; delivering feedback on participants' progress encourages GP support for social prescribing.[28 30 31]

Costs

The two comparative evaluations reported costs. One found total mean costs were greater in the intervention group (£153) compared with the control group (£133).[18] The other reported no statistically significant differences between the financial and environmental costs of healthcare use between the intervention and control groups[19].

One before and after study undertook a cost-benefit analysis using estimated input costs and benefits derived from 12 month outcome data obtained for 108 patients referred to social prescribing (42 of whom were referred to funded voluntary and community service providers). A total NHS cost reduction of £552,189 was generated by multiplying the estimated per-patient cost reduction by the total number of referrals (n=1118) to funded voluntary and community service providers of was achieved over the 2 year course of a social prescribing pilot programme. This estimate was compared with total estimated input costs of £1.1 million.[20]

For peer review only - http://bmjopen.bmj.com/site/about/guidelines.xhtml

One other report of an evaluation estimated total running costs of £83,144 for the programme for one year.[3]

Discussion

This systematic review has examined the evidence to inform the commissioning of social prescribing schemes. Overall, we identified 15 evaluations conducted in UK settings but have found little convincing evidence for either effectiveness or value for money.

Most of the evaluations of social prescribing activity are small scale and limited by poor design and reporting. Missing information has made it difficult to assess who received what, for what duration, with what effect and at what cost. Common design weaknesses include a lack of comparators (increasing the risk of bias), loss to follow up, short follow up durations and a lack of standardised and validated measuring tools. There is also a distinct failure to either consider and or adjust for potential confounding factors, undermining the ability to attribute any reported positive outcomes to the intervention (or indeed interventions) received. This is particularly important as most referred patients appear to have been receiving other interventions and so we have no way of assessing the relative contributions of the interventions to the outcomes reported. Despite these methodological shortcomings most evaluations have presented positive conclusions, generating a momentum for social prescribing that does not appear to be warranted.

Strengths and limitations

Our systematic review appears to be the first to assess the effectiveness of social prescribing programmes relevant to the UK NHS setting. We have searched for full publications and grey literature since 2000 but it is possible that we have not identified some local evaluations. Publication bias occurs when the results of published studies are systematically different from results of unpublished studies. However, we think it unlikely that any unidentified evaluations will be more robust than those included in the review.

Many of the evaluations presenting positive conclusions were written as descriptive reports with limited or no supporting data presented. As such, they did not adhere to formal reporting standards that would be expected in reports to funding agencies or in academic journal articles. This made extracting any relevant data difficult and it is possible information relevant to outcomes is missed. Even if this shortcoming of data completeness were to be addressed we believe that it would do little to alter the overall picture of a low quality evidence base with a high risk of bias.

For peer review only - http://bmjopen.bmj.com/site/about/guidelines.xhtml

For peer review only

Implications

Our systematic review has not established that there is clear evidence that social prescribing is ineffective. Rather, we are not yet able to reliably judge which if any social prescribing programmes demonstrate a degree of promise and so could be considered further. The use of a link worker is the key feature of social prescribing. How this link-worker role was fulfilled varied significantly between projects. So here again, we are not able to reliably judge the type of skills set or level of training and knowledge people require to effectively fulfil this role. For those seeking to commission new or extend existing schemes this evidence gap is a hindrance rather than a help, especially so given the widespread support and advocacy for social prescribing at the policy level.

Whilst the tension between rigour and 'good enough' evidence has long been recognised,[34] even 'good enough' is severely lacking from the social prescribing literature be that in the design or in the conduct of the evaluations themselves. This may in part reflect the way schemes have 'emerged' rather than being systematically planned with evaluation built in from the outset. Nevertheless, if social prescribing is to realise its potential then there is an urgent need to improve the ways by which schemes are evaluated.

Prospective pathways for undertaking rigorous planned experimental evaluation are well defined,[35] but the opportunity, time and resources needed to employ these in a service context can be limited. However, this does not serve as an excuse for inaction and in the current financial climate we should of course only be investing in those services where we can demonstrate real benefit over existing ways of working. What this should mean for future evaluation of social prescribing is that a more coordinated approach to the planning, implementation and evaluation of new and existing schemes is undertaken. This could and should involve the adoption of a common analytical framework which in turn will facilitate standardised metrics, cross-site comparison and shared learning. The IDEAL framework offers one such pathway to navigate the evaluation continuum that would allow for the iterative development and evaluation of whether social prescribing is likely to succeed in a particular setting and allow for adaptation, refinement and system integration without losing sight of the need for more rigorous testing before wider spread.[36] Whatever analytical framework is adopted, Lamont and colleagues[37] have proposed five essential questions for evaluation which those planning to undertake evaluations of social prescribing programmes would do well to heed. These are:

Why—Clarify aims and establish what we already know from evidence

Who—Identify and engage stakeholders and likely users of research at outset

For peer review only - http://bmjopen.bmj.com/site/about/guidelines.xhtml

How—Think about study design, using an appropriate mix of methods, and adjust for bias where possible (or at least acknowledge)

What—Consider what to measure (activity, costs, outcomes) and combine data from different sources

When— Pay attention to timing of results to maximise impact

Alongside these, we would also emphasise that that rigorous conduct and transparent reporting (regardless of 'success' or 'failure') are essential. Reporting guidelines such as SQUIRE[38] with its focus on explaining 'Why did you start?', 'What did you do?', 'What did you find?' and 'What does it mean? could readily be applied to ensure that learning is systematically captured in a generalisable format. This in turn would serve to ensure that any future decisions relating to the continuation or wider spread of social prescribing schemes are transparent and evidence informed.

Conclusions

Social prescribing is being widely advocated and implemented but current evidence fails to provide sufficient detail to judge either success or value for money. If social prescribing is to realise its potential, future evaluations must be comparative by design and consider when, by whom, for whom, how well and at what cost.

For peer review only

For peer review only - http://bmjopen.bmj.com/site/about/guidelines.xhtml

Contributors:

PMW took overall responsibility for the systematic review. LB, AB and PMW were involved in all stages of the review from development of the protocol, through screening studies and data extraction to analysis and synthesis and production of the final manuscript. KF provided input at all stages of the review and commented on drafts of the review. KW conducted literature searches and contributed to the methods section of the review. All authors approved the final version and PMW is the guarantor.

Funding:

This review was funded as part of research funded by the NIHR Health Services and Delivery Research programme (Project ref: 12/5002/18) with additional funding (for PMW) from the NIHR Collaboration for Leadership in Applied Health Research and Care (CLAHRC) Greater Manchester.

Disclaimer:

This report presents independent research funded by the National Institute for Health Research (NIHR). The views expressed are those of the authors and do not necessarily reflect those of the NIHR Health Services and Delivery Research programme, NIHR CLAHRC Greater Manchester or the Department of Health.

Data sharing

All available data can be obtained from the corresponding author.

For peer review only

References

1. NHS England. *Integrated Care Pioneer Programme: annual report 2014: pioneer profiles and case study examples*. Redditch: NHS England 2014.
2. Iniversity of Westminster. *Report of the annual Social Prescribing Network conference*. London: University of Westminster, 2016.
3. Kimberlee R, Ward R, Jones M, et al. *Measuring the economic impact of Wellspring Healthy Living Centre's Social Prescribing Wellbeing Programme for low level mental health issues encountered by GP services*. Bristol: University of the West of England, 2014.
4. Secretary of State for Health. *Saving Lives: Our Healthier Nation*. London: The Stationery Office, 1999.
5. Department of Health, *White paper. Our health, our care, our say: a new direction for community services*. Crown Copyright, 2006.
6. Matthews-King A. *GP leader appointed clinical champion for social prescribing*. Pulse. London: Cogora Limited, 30 June 2016.
7. Committee. H. *Oral evidence: Public expenditure on health and social care*, HC 679 Q652, 2014.
8. Wilson PM, Farley K, Thompson C, et al. Effects of a demand-led evidence briefing service on the uptake and use of research evidence by commissioners of health services: protocol for a controlled before and after study. *Implement Sci* 2015; 10:7.
9. Morton L, Ferguson M, Baty F. Improving wellbeing and self-efficacy by social prescription. *Pub Health* 2015; 129(3):286-9.
10. Jones M, Kimberlee R, Deave T, et al. The role of community centre-based arts, leisure and social activities in promoting adult well-being and healthy lifestyles. *Int J Environ Res Publ Health* 2013; 10(5):1948-62.
11. Orrow G, Kinmonth AL, Sanderson S, et al. Effectiveness of physical activity promotion based in primary care: systematic review and meta-analysis of randomised controlled trials. *BMJ* 2012;344:e1389 doi: doi: 10.1136/bmj.e1389
12. Pavey TG, Taylor AH, Fox KR, et al. Effect of exercise referral schemes in primary care on physical activity and improving health outcomes: systematic review and meta-analysis *BMJ* 2011; 343:d6462
13. Washburn RA, Lambourne K, Szabo AN, et al. Does increased prescribed exercise alter non-exercise physical activity/energy expenditure in healthy adults? A systematic review. *Clin Obes* 2013; 4:1-20.
14. Higgins JPT, Green S, (editors). *Cochrane Handbook for Systematic Reviews of Interventions Version 5.1.0*. The Cochrane Collaboration; 2011.
15. National Heart LaBI. *Quality Assessment Tool for Before-After (Pre-Post) Studies With No Control Group.* 2014. Available from: https://www.nhlbi.nih.gov/health-pro/guidelines/in-develop/cardiovascular-risk-reduction/tools/before-after [Date accessed 10 Nov 2016]
16. Kilgarriff-Foster A, Alicia OC. Exploring the components and impact of social prescribing. *J Public Ment Health* 2015;14(3):127-34.
17. Thomson LJ, Camic PM, Chatterjee HM. *Social Prescribing: A review of community referral schemes.* London: University College London, 2015.
18. Grant C, Goodenough T, Harvey I, et al. A randomized controlled trial and economic evaluation of a referrals facilitator between primary care and the voluntary sector. *BMJ* 2000; 320:419-23.
19. Maughan DL, Patel A, Parveen T, et al. Primary-care-based social prescribing for mental health: an analysis of financial and environmental sustainability. *Prim Health Care Res Dev* 2016; 17(2):114-21.
20. Dayson C, Bashir N. *The social and economic impact of the Rotherham Social Prescribing Pilot: Main Evaluation Report*. Centre for Regional Economic and Social Research, Sheffield Hallam University, 2014.
21. Friedli L, Themessl-Huber M, Butchart M. *Evaluation of Dundee Equally Well Sources of Support: Social Prescribing in Maryfield.* Evaluation Report Four, 2012.

For peer review only

For peer review only - http://bmjopen.bmj.com/site/about/guidelines.xhtml

22. Grayer J, Cape J, Orpwood L, et al. Facilitating access to voluntary and community services for patients with psychosocial problems: a before-after evaluation. *BMC Fam Pract* 2008; 9(27) doi: 10.1186/1471-2296/9/27
23. Faulkner M. Supporting the psychosocial needs of patients in general practice: the role of a voluntary referral service. *Patient Educ Couns* 2004; 52:41-46.
24. Age Concern Yorkshire and Humber. Social Prescribing. A model for partnership working between primary care and the voluntary sector. http://www.ageuk.org.uk/Documents/EN-GB/For-professionals/Health-and-wellbeing/Social_Prescribing_Report.pdf?dtrk=true 2012.
25. Baines A. *Rugby Social Prescribing Project ConnectWELL. Harnessing community capacity to improve health and wellbeing*. Roundberry Projects: Mid-term evaluation report, 2015.
26. ERS Research and Consultancy. *Newcastle Social Prescribing Project: final report*, 2013.
27. Longwill A. *Independent evaluation of Hackney WellFamily service: Improving Health and Wellbeing*, 2014.
28. South J, Higgins TJ, Woodall J, et al. Can social prescribing provide the missing link? *Prim Health Care Res Dev* 2008; 9(04):310-18.
29. White J, Kinsella K, South J. *An evaluation of social prescribing health trainers in south and west Bradford*, 2010.
30. Woodall J, South J. *The evaluation of the CHAT social prescribing scheme in Bradford South and West PCT*, 2005.
31. Involve North East. *Newcastle West Clinical Commissioning Group social prescribing pilot project: the views of participating health professionals*, 2013.
32. Brandling J, House W, Howitt D, et al. *New routes: pilot research project of a new social prescribing service provided in Keynsham*, 2011.
33. Cochrane Effective Practice and Organisation of Care Review Group (EPOC). *What study designs should be included in an EPOC review?* EPOC Resources for review authors. . Oslo: Norwegian Knowledge Centre for the Health Services, 2013.
34. Auerbach AD, Landefeld CS, Shojania KG. The tension between needing to improve care and knowing how to do it. *N Engl J Med* 2007; 357(6):608-13.
35. Craig P, Dieppe P, Macintyre S, et al. Developing and evaluating complex interventions: the new Medical Research Council guidance. *BMJ* 2008;337:a1655.
36. Wilson PM, Boaden R, Harvey G. Plans to accelerate innovation in health systems are less than IDEAL. *BMJ Qual Saf* 2015 doi: 10.1136/bmjqs-2015-004605
37. Lamont T, Barber N, de Pury J, et al. New approaches to evaluating complex health and care systems. *BMJ* 2016; 352:i154.
38. Ogrinc G, Davies L, Goodman D, et al. SQUIRE 2.0 (Standards for QUality Improvement Reporting Excellence): revised publication guidelines from a detailed consensus process. *BMJ Qual Saf* 2015; pii: bmjqs-2015-004411

For peer review only - http://bmjopen.bmj.com/site/about/guidelines.xhtml

Table 1: Characteristics of social prescribing project evaluations

Project information	Referral activity	Participants in evaluation (excluding health professionals and link-workers)	Facilitator/Co-ordinator skills and training	Activities patients referred to by Social Prescribing Facilitator/Coordinator
**Project name, location:** Amalthea project, Avon<br><br>**Author, year:** Grant, 2000<br><br>**Date project established (or time period of evaluation):** Aug 1997 to Sep 1998<br><br>**Type of evaluation:** Randomised controlled trial	**Referred to link-worker**: N=90<br><br>**Attended link-worker appointment:** 71/90 (79%)<br><br>**Attended a prescribed activity/services:** not reported<br><br>**GP surgeries involved:** N=26	**Approached to participate:** N=168<br><br>**Agreed to participate:** N=161 (90 randomised to intervention; 71 randomised to control)<br><br>Participants in the control group received routine care from their GP<br><br>**Included in evaluation analysis**: 69% of 90 for intervention an 67% of 71 for control followed up at 4 months	Three project facilitators from different backgrounds were trained and supervised by the organisation	Voluntary sector contacts available: National Schizophrenia Fellowship; Counselling on Alcohol and Drugs; Alcoholics Anonymous; Over Eaters Anonymous; Local eating disorders group; Triumph over Phobia; Womankind; Counselling Network; CRUSE; RELATE; Befrienders International; Local carer support group; Princess Royal Trust for Carers; Royal British Legion; Crisis; Migraine Trust; Local assertiveness training group; National Society for the Prevention of Cruelty to Children; Multiple Sclerosis Society; Disability Living Foundation; British Trust for Conservation Volunteers; Citizens Advice Bureau; Local meet a mum association; Local toddler group; Local social group for the elderly; University of the Third Age; Brunelcare; Battle against Tranquillisers; Women's Royal Voluntary Service
**Project name, location:** Connect project, Carlisle<br><br>**Author, year:** Maughan, 2016<br><br>**Date project established (or time period of evaluation):**Oct 2011 to Mar 2014<br><br>**Type of evaluation:** Controlled before and after study	**Referred to link-worker**: not reported<br><br>**Attended link-worker appointment:** N=30<br><br>**Attended a prescribed activity/services:** not reported<br><br>**GP surgeries involved:** N=1	**Approached to participate:** not reported<br><br>**Agreed to participate:** N=59 (30 in intervention group; 29 in control group)<br><br>Participants in the control group received routine care from their GP<br><br>**Included in evaluation analysis**: 28/30 (93%) in intervention; 29/29 (100%) in control	Non-healthcare staff, provided with brief training about local services, completing questionnaires and managing risk.<br><br>Not reported	Available services across third, public and private sectors, self-help, self-management resources, educational, leisure and recreational facilities and fitness-, health- and exercise-related activities. Example given: The Eden Timebank a skills exchange and social network where members earn credits for helping another member or the wider community.
**Project name, location:** Rotherham Social Prescribing project<br><br>**Author, year:** Dayson, 2014<br><br>**Date project**	**Referred to link-worker**: N=1607<br><br>**Attended link-worker appointment:** not reported<br><br>**Attended a prescribed**	**Approached to participate:** not reported<br><br>**Agreed to participate:** not reported<br><br>**Included in evaluation analysis**:	Not reported.	Information and advice; community activity; physical activity; befriending and enabling

For peer review only - http://bmjopen.bmj.com/site/about/guidelines.xhtml

**established (or time period of evaluation):** Apr 2012 to Mar 2014<br><br>**Type of evaluation:** Uncontrolled before and after study	**activity/services:** not reported (1118 people were referred onwards to other funded voluntary and community sector services)<br><br>**GP surgeries involved:** N=29	i. Hospital episode data analysis: N=451 followed up at 6 months; N=108 followed at 12 months (of which n=42 referred on to a funded voluntary and community service provider)<br>ii. Wellbeing outcomes analysis: 280/819 followed up at 3-4 months		
**Project name, location:** Dundee Equally Well Sources of Support<br><br>**Author, year:** Friedli, 2012<br><br>**Date project established (or time period of evaluation):** Mar 2011 to Jun 2012<br><br>**Type of evaluation:** Uncontrolled before and after study	**Referred to link-worker**: N=123<br><br>**Attended link-worker appointment:** 61/123 (50%)<br><br>**Attended a prescribed activity/services:** 26 out of 26 referred to an activity attended that activity (119 link-worker referrals were made into 47 different community services or groups)<br><br>**GP surgeries involved:** N=1	**Approached to participate:** not reported<br><br>**Agreed to participate:** not reported<br><br>**Included in evaluation analysis**: N=16	Not reported.	Community based information, support and/or activities
**Project name, location:** Graduate Primary Care Mental Health Worker Community Link Scheme, north London<br><br>**Author, year:** Grayer, 2008<br><br>**Date project established (or time period of evaluation):**NR<br><br>**Type of evaluation:** Uncontrolled before and after study	**Referred to link-worker**: N=255<br><br>**Attended link-worker appointment:** N=151<br><br>**Attended a prescribed activity/services:** 58% attended at least one of the services suggested<br><br>**GP surgeries involved:** N=13	**Approached to participate:** N=151<br><br>**Agreed to participate:** 108/151<br><br>**Included in evaluation analysis**: N=75/108 followed up at 3 months	Psychology graduates with some voluntary clinical experience but no formal mental health training. In-house training and supervision from two clinical psychologists.<br><br>Not reported.	Community resources identified through searches of paper and electronic directories, telephone enquiries, and other sources.
**Project name, location:** Wellbeing Programme at Wellspring Healthy Living Centre, Bristol<br><br>**Author, year:** Kimberlee, 2014<br><br>**Date project established (or time period of evaluation):** May 2012 to Apr 2013	**Referred to link-worker**: Unclear<br><br>**Attended link-worker appointment:** N=128<br><br>**Attended a prescribed activity/services:** not reported<br><br>**GP surgeries involved:** not reported	**Approached to participate:** N=128<br><br>**Agreed to participate:** N=128<br><br>**Included in evaluation analysis**:<br>i. Health and wellbeing outcomes N=70 followed up at 3 months<br>ii. GP attendance data N=40 12 months before	Not reported	Peer support groups, creative arts, physical activities, cooking courses, complementary therapies

For peer review only - http://bmjopen.bmj.com/site/about/guidelines.xhtml

**Type of evaluation:** Uncontrolled before and after study		and after baseline		
**Project name, location:** Age Concern, Yorkshire & Humber<br><br>**Author, year:** Age Concern, 2012<br><br>**Date project established (or time period of evaluation):** Apr 2011 to Sep 2011<br><br>**Type of evaluation:** Descriptive report	**Referred to link-worker**: N=55<br><br>**Attended link-worker appointment:** not reported<br><br>**Attended a prescribed activity/services:** not reported<br><br>**GP surgeries involved:** N=12	**Approached to participate:** unclear<br><br>**Agreed to participate:** unclear<br><br>**Included in evaluation analysis**: not reported	A skilled member of Age UK staff	Age UK services including: befriending, day clubs, luncheon clubs, information and advice, benefit checks, trips, theatre outings, computer training, advocacy, legal advice, will-writing service, volunteering, Fit as a Fiddle classes, art groups, memory loss services
**Project name, location:** ConnectWell, Coventry<br><br>**Author, year:** Baines, 2015<br><br>**Date project established (or time period of evaluation):** Aug 2014 to Aug 2015<br><br>**Type of evaluation:** Descriptive report (with qualitative element)	**Referred to link-worker**: N=39<br><br>**Attended link-worker appointment:** 24/39 (62%)<br><br>**Attended a prescribed activity/services:** not reported<br><br>**GP surgeries involved:** N=4	**Approached to participate:** not reported<br><br>**Agreed to participate:** not reported<br><br>**Included in evaluation analysis**: N=5	Volunteers attend group training session then inductions for specific role. Additional training offered e.g. mentoring, dementia awareness. Supervised by WCAVA	Befriending, lunch club, advice & information services, housing/homelessness services, counselling, sport, art, volunteering, support group, social activities
**Project name, location:** Newcastle Social Prescribing Project<br><br>**Author, year:** ERS Research and Consultancy, 2013 Involve North East, 2013<br><br>**Date project established (or time period of evaluation):** Jan 2012 to Mar 2013<br><br>**Type of evaluation:** 2 Descriptive reports (one with qualitative element)	**Referred to link-worker**: N=124<br><br>**Attended link-worker appointment:** 87/124 (70%)<br><br>**Attended a prescribed activity/services:** not reported<br><br>**GP surgeries involved:** N=6	**Approached to participate:** not reported<br><br>**Agreed to participate:** not reported<br><br>**Included in evaluation analysis**: N=9	Existing staff member in each VCSO with knowledge of local community and services, LTCs. Skills and attributes specified.	Support with personalized goal setting and buddying, self care, and signposting to information, advice and support through an agency: Age UK; HealthWORKS; Newcastle Carers; Search; West End Befrienders
**Project name, location:** CHAT, south and west Bradford<br><br>**Author, year:** Woodall, 2005<br><br>**Date project established (or time period of evaluation):** Established 2004 Piloted Jan 2005 to Sep 2005	**Referred to link-worker**: N=81<br><br>**Attended link-worker appointment:** not reported<br><br>**Attended a prescribed activity/services:** not reported<br><br>**GP surgeries involved:** N=3	**Approached to participate:** not reported<br><br>**Agreed to participate:** not reported<br><br>**Included in evaluation analysis**: N=10	Non-clinical Health Trainers, a public health workforce supported by the DH	Local community and voluntary services.

For peer review only - http://bmjopen.bmj.com/site/about/guidelines.xhtml

**Type of evaluation:** Descriptive report (with qualitative element)				
**Project name, location:** CHAT, south and west Bradford<br><br>**Author, year:** South, 2008<br><br>**Date project established (or time period of evaluation):** May 2005 to Oct 2006<br><br>**Type of evaluation:** Qualitative study	**Referred to link-worker**: N=223<br><br>**Attended link-worker appointment:** not reported<br><br>**Attended a prescribed activity/services:** not reported<br><br>**GP surgeries involved:** not reported	**Approached to participate:** not reported<br><br>**Agreed to participate:** not reported<br><br>**Included in evaluation analysis**: N=10	Non-clinical Health Trainers, a public health workforce supported by the DH	Community and voluntary sector groups and services such as: Luncheon clubs; Befriending groups; Social services; Volunteering organizations; Getting back into work groups; Literacy classes; Debt advice; Access bus; Bereavement groups; Reminiscing groups; Arts and craft groups; Music groups
**Project name, location:** Health Trainer and Social Prescribing Service, south and west Bradford<br><br>**Author, year:** White 2010<br><br>**Date project established (or time period of evaluation):** Established 2006 (evolved from CHAT) Jan 2010 to Sep 2010<br><br>**Type of evaluation:** Descriptive report (with qualitative element)	**Referred to link-worker**: N=484<br><br>**Attended link-worker appointment:** not reported<br><br>**Attended a prescribed activity/services:** not reported<br><br>**GP surgeries involved:** N=21	**Approached to participate:** not reported<br><br>**Agreed to participate:** not reported<br><br>**Included in evaluation analysis**: N=12	Non-clinical Health Trainers, a public health workforce supported by the DH	Local voluntary and community sector social groups and support agencies.<br><br>Health trainer can develop personal health action plan.
**Project name, location:** Doncaster Patient Support Service<br><br>**Author, year:** Faulkner, 2004<br><br>**Date project established (or time period of evaluation):** April 2001 to February 2002<br><br>**Type of evaluation:** Qualitative study	**Referred to link-worker**: 200<br><br>**Attended link-worker appointment:** N=132<br><br>**Attended a prescribed activity/services:**Not reported<br><br>**GP surgeries involved:** N=1	**Approached to participate:** 17 patients and 9 volunteers<br><br>**Agreed to participate:** Patients: N=11 Volunteers: N=9<br><br>**Included in evaluation analysis**: Patients: N=11 Volunteers: N=9	Volunteers given 3 day training including basic counselling knowledge and skills, team building strategies, and visits from community services they might refer people to. Ongoing training and supervision provided.	Facilitated access to services providing: advice on disability services, advice on nursing homes; alcohol support; benefit issues; family/matrimonial support; family support for drug users; advice on housing/social services; legal issues (e.g. The Women's Centre; Mind; Relate; Alcohol and Drug Advice)
**Project name, location:** WellFamily service in Hackney*<br><br>**Author, year:** Longwill, 2014<br><br>**Date project established (or time period of evaluation):**	**Referred to link-worker**: N=1466<br><br>**Attended link-worker appointment:** N=1089<br><br>**Attended a prescribed activity/services:** N=712	**Approached to participate:** Not reported<br><br>**Agreed to participate:** Not reported<br><br>**Included in evaluation analysis**: GAD7, PHQ9: N=387	Family action workers and senior practitioners with a variety of skills and experience. Some with undergraduate and postgraduate qualifications in counselling, group therapy, medicine	Short term counselling, advice and practical support. Local voluntary, community, and social enterprise sector services.<br><br>Other social and health services such as debt counselling, housing departments and health

For peer review only - http://bmjopen.bmj.com/site/about/guidelines.xhtml

First established 1996; Period of evaluation: 2012-13<br><br>**Type of evaluation:** Descriptive report (with qualitative element)	**GP surgeries involved:** 32	Patient survey: N=92 respondents (out of active caseload of approx. 120)<br>GP survey: N=27 respondents (out of 160 surveyed GPs)	and psychotherapy.<br><br>Family Action counsellors - professionally qualified and under regular supervision	services
**Project name, location:** 'New Routes', Keynsham (Bath and North East Somerset)<br><br>**Author, year:** Brandling, 2011<br><br>**Date project established (or time period of evaluation):**2-year pilot established October 2009<br><br>**Type of evaluation:** Descriptive report (with qualitative element)	**Referred to link-worker**: N=90<br><br>**Attended link-worker appointment:** not reported<br><br>**Attended a prescribed activity/services:** N=42<br><br>**GP surgeries involved:** 3	**Approached to participate:** Not reported<br><br>**Agreed to participate:** Not reported<br><br>**Included in evaluation analysis**: WEMWBS completed at 6-12 months N=7<br>MYMOP2 completed at 6-12 months N=12<br><br>Qualitative interviews N=21	Co-ordinators role modelled on Amalthea project[13]<br><br>Skills and training not reported	46 different types of organizations and activities were part of the pilot.<br><br>Most popular activities: volunteering; befriending; walking groups; art groups

NR, not reported; WEMWBS, Warwick Edinburgh Mental Wellbeing Scale; MYMOP2, Measure Yourself Medical Outcome Profile

Table 2: Quality assessment and risk of bias

**Comparative evaluations**			
**Study**	**Quality criteria**	**Risk of bias**	**Notes**
**Grant 2000**<br><br>**RCT**	Sequence generation	Low	Sealed opaque envelopes prepared by research team. Stratification by practice and blocks of six used (3 intervention/3 control).
	Allocation concealment	Unclear	Sequentially numbered envelopes opened. In two practices there was evidence that the randomization process was initially misunderstood: six patients excluded.
	Blinding of participants and personal	Not possible	
	Blinding of outcome assessment	Unclear	
	Incomplete outcome data	High	32% loss to follow-up at 4 months
	Selective outcome reporting	Unclear	
	Other potential threats to validity	Unclear	Numbers potentially eligible but not recruited unknown<br>Recruited general practices were not a random sample: participating doctors were likely to be more<br>interested in the research question and may have managed psychosocial problems more actively, which could have diminished reported estimates

For peer review only - http://bmjopen.bmj.com/site/about/guidelines.xhtml

			of effects
**Maughan 2016**<br><br>**CBA**	Is there a suitable comparison group?	Yes	One intervention and one control group, drawn from the same general practice with similar patient characteristics.<br>Models environmental costs (in terms of carbon footprint)<br>Data were retrospectively collected from GP health records for a two-year period.<br>Two participants in intervention group excluded from analysis<br>Financial and environmental impacts calculated for each outcome using national averages or accepted conversion factors
	Do the authors use theory to underpin the project/evaluation?	No	
	Were appropriate methods used for data collection and analysis?	Yes	
	Were efforts made to assess patient experience?	No	
**Uncontrolled before and after evaluations**			
**Study**	**Quality criteria**	**Judgement**	**Notes**
**Dayson 2014**	Was the study question or objective clearly stated?	Yes	Small sample of those referred (N=1607) participated in evaluation – HES data at 6 months N=451, at 12 months N=108; wellbeing data at 3-4 months 280/819<br><br>Methods of qualitative analysis of patient experience unclear
	Were eligibility/selection criteria for the study population prespecified and clearly described?	Not reported	
	Were the participants in the study representative of those who would be eligible for the test/service/intervention in the general or clinical population of interest?	Yes	
	Were all eligible participants that met the prespecified entry criteria enrolled?	Not reported	
	Was the sample size sufficiently large to provide confidence in the findings?	No	
	Was the test/service/intervention clearly described and delivered consistently across the study population?	Not reported	
	Were the outcome measures prespecified, clearly defined, valid, reliable, and assessed consistently across all study participants?	Yes	
	Were the people assessing the outcomes blinded to the participants' exposures/interventions?	Not reported	
	Was the loss to follow-up after baseline 20% or less? Were those lost to follow-up accounted for in the analysis?	No	
	Did the statistical methods examine changes in outcome measures from before to after the intervention? Were statistical tests done that provided p values for the pre-to-post changes?	Yes	
	Were outcome measures of interest taken multiple times before the	No	

For peer review only - http://bmjopen.bmj.com/site/about/guidelines.xhtml

	intervention and multiple times after the intervention (i.e., did they use an interrupted time-series design)?		
	If the intervention was conducted at a group level (e.g., a whole hospital, a community, etc.) did the statistical analysis take into account the use of individual-level data to determine effects at the group level?	Not applicable	
**Friedli 2012**	Was the study question or objective clearly stated?	Yes	Details of pre and post intervention outcomes not reported<br>Small sample size<br>Timing of post intervention assessment not reported<br>Methods of qualitative analysis of patient and provider/referrer experience unclear
	Were eligibility/selection criteria for the study population prespecified and clearly described?	No	
	Were the participants in the study representative of those who would be eligible for the test/service/intervention in the general or clinical population of interest?	Yes	
	Were all eligible participants that met the prespecified entry criteria enrolled?	Not applicable	
	Was the sample size sufficiently large to provide confidence in the findings?	No	
	Was the test/service/intervention clearly described and delivered consistently across the study population?	Not reported	
	Were the outcome measures prespecified, clearly defined, valid, reliable, and assessed consistently across all study participants?	No	
	Were the people assessing the outcomes blinded to the participants' exposures/interventions?	No	
	Was the loss to follow-up after baseline 20% or less? Were those lost to follow-up accounted for in the analysis?	No	
	Did the statistical methods examine changes in outcome measures from before to after the intervention? Were statistical tests done that provided p values for the pre-to-post changes?	No	
	Were outcome measures of interest taken multiple times before the intervention and multiple times after the intervention (i.e., did they use an interrupted time-series design)?	No	
	If the intervention was conducted at a group level (e.g., a whole hospital, a community, etc.) did the statistical	Not applicable	

For peer review only - http://bmjopen.bmj.com/site/about/guidelines.xhtml

	analysis take into account the use of individual-level data to determine effects at the group level?		
**Grayer 2008**	Was the study question or objective clearly stated?	Yes	GP practices volunteered and may not be representative of practices overall<br>Patients who consented to participate in evaluation were more likely to speak English as a first language than those who did not consent<br>No significant differences at baseline between those successfully followed up and those lost to follow up<br>95% confidence intervals (no P values) reported for changes in GHQ-12, CORE-OM and WSAS scores
	Were eligibility/selection criteria for the study population prespecified and clearly described?	Yes	
	Were the participants in the study representative of those who would be eligible for the test/service/intervention in the general or clinical population of interest?	Yes	
	Were all eligible participants that met the prespecified entry criteria enrolled?	No	
	Was the sample size sufficiently large to provide confidence in the findings?	No	
	Was the test/service/intervention clearly described and delivered consistently across the study population?	Yes	
	Were the outcome measures prespecified, clearly defined, valid, reliable, and assessed consistently across all study participants?	Yes	
	Were the people assessing the outcomes blinded to the participants' exposures/interventions?	Not reported	
	Was the loss to follow-up after baseline 20% or less? Were those lost to follow-up accounted for in the analysis?	No	
	Did the statistical methods examine changes in outcome measures from before to after the intervention? Were statistical tests done that provided p values for the pre-to-post changes?	Yes	
	Were outcome measures of interest taken multiple times before the intervention and multiple times after the intervention (i.e., did they use an interrupted time-series design)?	No	
	If the intervention was conducted at a group level (e.g., a whole hospital, a community, etc.) did the statistical analysis take into account the use of individual-level data to determine effects at the group level?	Not applicable	
**Kimberlee 2014**	Was the study question or objective clearly stated?	Yes	SROI analysis presents data for all baseline completers and the smaller percentage who were followed up; possible bias towards positive finding
	Were eligibility/selection criteria for	No	

For peer review only

	the study population prespecified and clearly described?		for intervention<br><br>Unclear whether calculations of mean differences in scale scores used all baseline data or baseline data for follow up completers only<br><br>P values reported for change from baseline at 3 months in PHQ-9 depression scores
	Were the participants in the study representative of those who would be eligible for the test/service/intervention in the general or clinical population of interest?	Yes	
	Were all eligible participants that met the prespecified entry criteria enrolled?	Not applicable	
	Was the sample size sufficiently large to provide confidence in the findings?	No	
	Was the test/service/intervention clearly described and delivered consistently across the study population?	Not reported	
	Were the outcome measures prespecified, clearly defined, valid, reliable, and assessed consistently across all study participants?	Yes	
	Were the people assessing the outcomes blinded to the participants' exposures/interventions?	Not reported	
	Was the loss to follow-up after baseline 20% or less? Were those lost to follow-up accounted for in the analysis?	No	
	Did the statistical methods examine changes in outcome measures from before to after the intervention? Were statistical tests done that provided p values for the pre-to-post changes?	Yes	
	Were outcome measures of interest taken multiple times before the intervention and multiple times after the intervention (i.e., did they use an interrupted time-series design)?	No	
	If the intervention was conducted at a group level (e.g., a whole hospital, a community, etc.) did the statistical analysis take into account the use of individual-level data to determine effects at the group level?	Not applicable	

For peer review only

For peer review only - http://bmjopen.bmj.com/site/about/guidelines.xhtml

Table 3: Health and wellbeing outcomes (validated measures)

Study (timing of outcome measure-ment post baseline measure-ment)	WEMWBS	HADS	GAD-7	PHQ-9	CORE-OM	WSAS	GHQ-12	COOP/WONCA
**RCTs**								
**Grant 2000 (4 months)**		Intervention group (N=62)* greater improvement than control group (N=48)*						Intervention group (N=62)* greater improvement than control group (N=48)*
**Before and after evaluations**								
**Friedli 2012 (NR)**	"Statistically significant improvement" in mental wellbeing (N=16) (scores not reported)					"Statistically significant improvement" in functional ability (N=16)(scores not reported)		
**Grayer 2008 (3 months)**					Small reduction in patients categorised as cases (N=74)	Improvement in work and social adjustment (N=69)	Four-fifths were cases at baseline, reducing to half of post intervention N=69)	
**Descriptive reports**								
**ERS Research and Consultancy 2013 (NR)**	Increase in mean score from 22 to 26 (N=16)							
**Longwill 2014 (NR)**			2.5 point reduction in score (P<0.001) (N=387)	3.1 point reduction in score (P<0.001) (N=387)				
**Brandling 2011 (6-12 months)**	“General positive trend but owing to low number of participants completing questionnaires no							

For peer review only - http://bmjopen.bmj.com/site/about/guidelines.xhtml

	further conclusions can be made"							

*calculated from reported percentage followed up at 4 months

WEMWBS: Warwick-Edinburgh Mental Wellbeing Scale; HADS: Hospital Anxiety and Depression Scale; GAD-7: General Anxiety Disorder-7; PHQ-9: Patient Health Questionnaire-9; CORE-OM: Core Outcome Measure; WSAS: Work and Social Adjustment Scale; GHQ-12: General Health Questionnaire-12; COOP/WONCA: Dartmouth COOP Functional Health Assessment Charts

For peer review only

For peer review only - http://bmjopen.bmj.com/site/about/guidelines.xhtml

Figure 1: PRISMA Flow Diagram

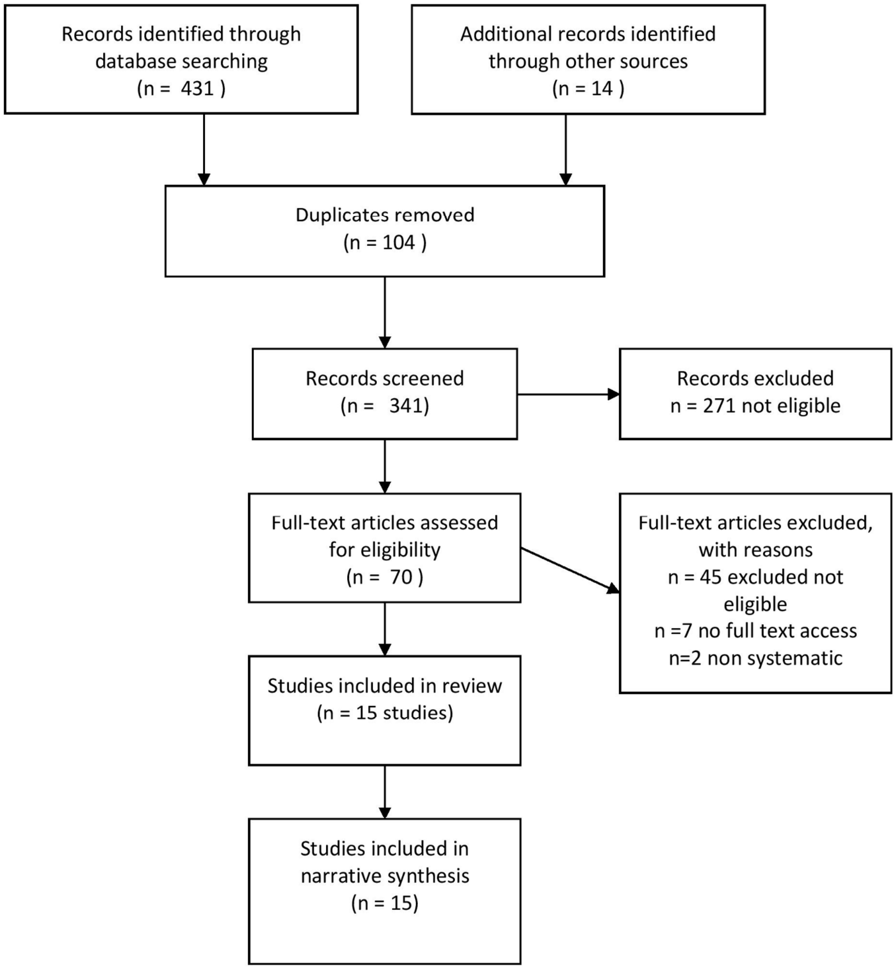

174x210mm (300 x 300 DPI)

Appendix 1: Search strategies

ASSIA via Proquest Search date 26th June 2015 and 5th February 2016

"social prescrib*" OR "social prescrip*" OR "community referral*"

CINAHL via EBSCO search date 26th June 2015 and 5th February 2016

social prescribing OR "social prescrip*" OR "community referral*"

For peer review only

Ovid MEDLINE(R) In-Process & Other Non-Indexed Citations and Ovid MEDLINE(R) <1946 to Present> searched 26th June 2015 and 5th February 2016

1 social prescrib$.ti,ab.
2 social prescrip$.ti,ab.
3 community referral$.ti,ab.
4 non-medical referral$.ti,ab.
5 well being program$.ti,ab.
6 well-being program$.ti,ab.
7 wellbeing program$.ti,ab.
8 1 or 2 or 3 or 4 or 5 or 6 or 7

Social Care Online via http://www.scie-socialcareonline.org.uk/ searched 26th June 2015 and 5th February 2016

“Social prescribing” OR “social prescription*” or “community referral*”

Social Policy & Practice via OVID search date 26th June 2015 and 5th February 2016

1 social prescrib$.ti,ab.
2 social prescrip$.ti,ab.
3 community referral$.ti,ab.
4 non-medical referral$.ti,ab.
5 well being program$.ti,ab.
6 well-being program$.ti,ab.
7 wellbeing program$.ti,ab.
8 1 or 2 or 3 or 4 or 5 or 6 or 7

Google search last performed 5th January 2016

Two reviewers independently searched google.co.uk using the search terms “social prescribing” and “community referral” and reviewed the search results from the first 10 pages

For peer review only - http://bmjopen.bmj.com/site/about/guidelines.xhtml

PRISMA 2009 Checklist

Section/topic	#	Checklist item	Reported on page #
**TITLE**			
Title	1	Identify the report as a systematic review, meta-analysis, or both.	1
**ABSTRACT**			
Structured summary	2	Provide a structured summary including, as applicable: background; objectives; data sources; study eligibility criteria, participants, and interventions; study appraisal and synthesis methods; results; limitations; conclusions and implications of key findings; systematic review registration number.	2
**INTRODUCTION**			
Rationale	3	Describe the rationale for the review in the context of what is already known.	4
Objectives	4	Provide an explicit statement of questions being addressed with reference to participants, interventions, comparisons, outcomes, and study design (PICOS).	4
**METHODS**			
Protocol and registration	5	Indicate if a review protocol exists, if and where it can be accessed (e.g., Web address), and, if available, provide registration information including registration number.	2
Eligibility criteria	6	Specify study characteristics (e.g., PICOS, length of follow-up) and report characteristics (e.g., years considered, language, publication status) used as criteria for eligibility, giving rationale.	5
Information sources	7	Describe all information sources (e.g., databases with dates of coverage, contact with study authors to identify additional studies) in the search and date last searched.	5
Search	8	Present full electronic search strategy for at least one database, including any limits used, such that it could be repeated.	Supp File
Study selection	9	State the process for selecting studies (i.e., screening, eligibility, included in systematic review, and, if applicable, included in the meta-analysis).	5-6
Data collection process	10	Describe method of data extraction from reports (e.g., piloted forms, independently, in duplicate) and any processes for obtaining and confirming data from investigators.	6
Data items	11	List and define all variables for which data were sought (e.g., PICOS, funding sources) and any assumptions and simplifications made.	5-6
Risk of bias in individual studies	12	Describe methods used for assessing risk of bias of individual studies (including specification of whether this was done at the study or outcome level), and how this information is to be used in any data synthesis.	6
Summary measures	13	State the principal summary measures (e.g., risk ratio, difference in means).	n/a
Synthesis of results	14	Describe the methods of handling data and combining results of studies, if done, including measures of consistency (e.g., I^2) for each meta-analysis.	n/a

For peer review only - http://bmjopen.bmj.com/site/about/guidelines.xhtml

PRISMA 2009 Checklist

Section/topic	#	Checklist item	Reported on page #
Risk of bias across studies	15	Specify any assessment of risk of bias that may affect the cumulative evidence (e.g., publication bias, selective reporting within studies).	n/a
Additional analyses	16	Describe methods of additional analyses (e.g., sensitivity or subgroup analyses, meta-regression), if done, indicating which were pre-specified.	n/a
**RESULTS**			
Study selection	17	Give numbers of studies screened, assessed for eligibility, and included in the review, with reasons for exclusions at each stage, ideally with a flow diagram.	7
Study characteristics	18	For each study, present characteristics for which data were extracted (e.g., study size, PICOS, follow-up period) and provide the citations.	17-23
Risk of bias within studies	19	Present data on risk of bias of each study and, if available, any outcome level assessment (see item 12).	8
Results of individual studies	20	For all outcomes considered (benefits or harms), present, for each study: (a) simple summary data for each intervention group (b) effect estimates and confidence intervals, ideally with a forest plot.	29
Synthesis of results	21	Present results of each meta-analysis done, including confidence intervals and measures of consistency.	n/a
Risk of bias across studies	22	Present results of any assessment of risk of bias across studies (see Item 15).	8
Additional analysis	23	Give results of additional analyses, if done (e.g., sensitivity or subgroup analyses, meta-regression [see Item 16]).	n/a
**DISCUSSION**			
Summary of evidence	24	Summarize the main findings including the strength of evidence for each main outcome; consider their relevance to key groups (e.g., healthcare providers, users, and policy makers).	11
Limitations	25	Discuss limitations at study and outcome level (e.g., risk of bias), and at review-level (e.g., incomplete retrieval of identified research, reporting bias).	11
Conclusions	26	Provide a general interpretation of the results in the context of other evidence, and implications for future research.	13
**FUNDING**			
Funding	27	Describe sources of funding for the systematic review and other support (e.g., supply of data); role of funders for the systematic review.	14

From: Moher D, Liberati A, Tetzlaff J, Altman DG, The PRISMA Group (2009). Preferred Reporting Items for Systematic Reviews and Meta-Analyses: The PRISMA Statement. PLoS Med 6(7): e1000097. doi:10.1371/journal.pmed1000097

For more information, visit: **www.prisma-statement.org**.

For peer review only - http://bmjopen.bmj.com/site/about/guidelines.xhtml
